# Modeling Meiotic Chromosomes Indicates a Size Dependent Contribution of Telomere Clustering and Chromosome Rigidity to Homologue Juxtaposition

**DOI:** 10.1371/journal.pcbi.1002496

**Published:** 2012-05-03

**Authors:** Christopher A. Penfold, Paul E. Brown, Neil D. Lawrence, Alastair S. H. Goldman

**Affiliations:** 1 Department of Molecular Biology and Biotechnology, Krebs Institute, The University of Sheffield, Sheffield, United Kingdom; 2 Department of Computer Science, The University of Sheffield, Sheffield, United Kingdom; 3 Sheffield Institute of Translational Neuroscience, The University of Sheffield, Sheffield, United Kingdom; 4 Systems Biology Centre, University of Warwick, Coventry, United Kingdom; Accelrys, United States of America

## Abstract

Meiosis is the cell division that halves the genetic component of diploid cells to form gametes or spores. To achieve this, meiotic cells undergo a radical spatial reorganisation of chromosomes. This reorganisation is a prerequisite for the pairing of parental homologous chromosomes and the reductional division, which halves the number of chromosomes in daughter cells. Of particular note is the change from a centromere clustered layout (Rabl configuration) to a telomere clustered conformation (bouquet stage). The contribution of the bouquet structure to homologous chromosome pairing is uncertain. We have developed a new *in silico* model to represent the chromosomes of *Saccharomyces cerevisiae* in space, based on a worm-like chain model constrained by attachment to the nuclear envelope and clustering forces. We have asked how these constraints could influence chromosome layout, with particular regard to the juxtaposition of homologous chromosomes and potential nonallelic, ectopic, interactions. The data support the view that the bouquet may be sufficient to bring short chromosomes together, but the contribution to long chromosomes is less. We also find that persistence length is critical to how much influence the bouquet structure could have, both on pairing of homologues and avoiding contacts with heterologues. This work represents an important development in computer modeling of chromosomes, and suggests new explanations for why elucidating the functional significance of the bouquet by genetics has been so difficult.

## Author Summary

Organisms store their genetic material in the form of chromosomes that must be replicated and shared out during cell division. In sexual reproduction the cell division, called meiosis, halves the number of chromosomes to form gametes. This halving requires a complex reorganisation of chromosomes. Each gamete receives one maternal or one paternal copy of every chromosome. This requires a pairing process between the maternal and paternal chromosomes of each type. Once paired the two chromosomes are organised in space to bias subsequent movement in opposite directions when the nucleus divides. How chromosomes pair is of great importance to understanding fertility, and manipulating chromosomes in crops species, for which it is desirable to breed in new genes to improve hardiness or yield. We have modelled chromosomes in 3-dimensions based on the experimental organism *Saccharomyces cerevisiae*. We used our model to ask if various physical features of chromosomes might influence their ability to pair. We found that binding chromosome ends to the nuclear wall and pushing those ends together helps to encourage pairing along the length of chromosomes. It has long been known this special chromosome organisation occurs in live cells, but the significance of it has been difficult to determine.

## Introduction

### Meiosis is a specialised form of cell division used by eukaryotes during sexual reproduction to produce gametes or spores

**Figure 1:**
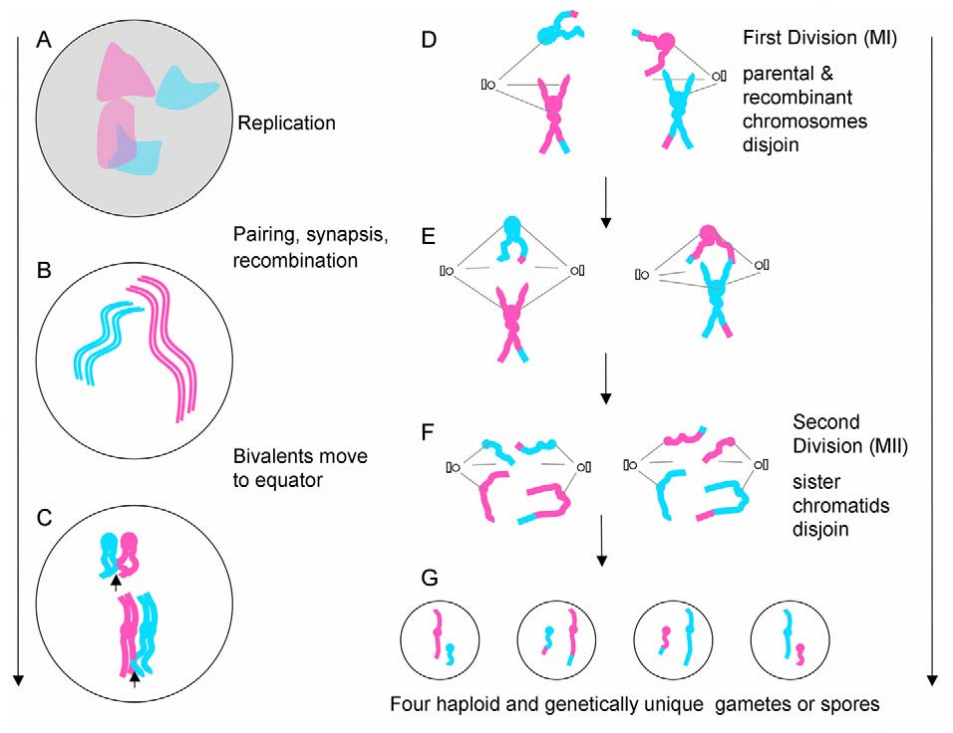
Meiosis is a form a cell division that produces four genetically unique haploid daughter cells. Meiosis starts of with DNA replication when (A) the chromosomes are relatively diffuse; two homologue pairs are shown in pink and blue (for different parental origin) in a background of other chromosomes, grey. (B) Following replication each chromosome now consists of two sister chromatids, which in most organisms condense and in all organisms homologous (maternal and paternal) chromosomes pair up. In most organisms the closest pairing confirmation leads to synapsis when protein structure called the synaptonemal complex holds homologues very close to each other (not shown). The process of recombination initiates during early chromosome pairing and is completed at the end of synapsis [88], [89]. (C) Homologues start to move apart and congress on the equator of the nucleus. They remain attached to each other due to the presence of crossovers and sister chromatids being held tightly together. (D) After attachment to the spindles the now recombinant homologous chromosomes are separated during Anaphase I. (E) A second spindle is built and (F) sister chromatids segregate during Anaphase II, yielding (G) four unique haploid gametes or spores.

There are two major forms of cell division among eukaryotes. Mitosis is used for cell duplication and meiosis is used to produce gametes and spores (Fig. 1). Meiosis is preceded, like mitosis, with a round of DNA synthesis that replicates all chromosomes. A major difference between these methods of cell division resides in the number of nuclear (and chromosomal) divisions. In mitosis there is a single nuclear division, restoring the normal chromosome complement in two daughter cells. In meiosis there are two rounds of nuclear division creating four daughter cells, with half the chromosome complement (Fig. 1 D to 1 G; review [1]). The sexual life cycle is completed when two of these haploid gametes, or spores, fuse to rebuild a diploid cell.

Each of the four gametes/spores produced during meiosis are genetically unique. During cell division allele combinations are assorted through two mechanisms. The first mechanism involves recombination between parental copies of each chromosome, brought about by a process called crossing-over (Fig. 1 C; arrows). Recombination occurs before the first division, so after division chromosomes become a patchwork mixture of paternal and maternal DNA (Fig. 1 D). The second mechanism comes from two rounds of independent assortment of chromosomes. During the first meiotic division one copy of each chromosome segregates away from its homologous partner, and there is no link between unrelated chromosome pairs (Fig. 1 D). During the second meiotic division sister chromatids segregate in opposite directions, and again there is no link in the direction of segregation for unrelated chromosomes (Fig. 1 F).

### To successfully complete the first meiotic division, chromosomes must undertake a dramatic program of reorganisation

Prior to meiosis the parental pair of homologous chromosomes are relatively dispersed throughout the nucleus (Fig. 1 A). In order that they become close enough to form crossovers, and then segregate from each other, homologues line up in pairs (reviews [1], [2]). Reorganising premeiotic chromosomes into ordered pairs involves chromosome condensation and movement that ultimately leads to their synapsis (Fig. 1 B). Studies mainly from budding yeast, *Saccharomyces cerevisiae*, show that precursor molecular events to crossing-over are concomitant with and probably part of the pairing process [1]. Once chromosomes are brought close enough they synapse, and subsequently mature crossover products can be detected in molecular assays [1]. Many genes are involved in causing and regulating the chromosome movements and crossing-over, and chromosome architecture itself plays a role in this physical process [3].

**Figure 2:**
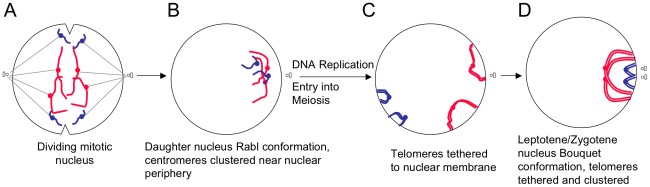
Chromosomes reorganise early in meiosis to form the bouquet structure. (A) During mitosis the nucleus divides and centromeres move towards the microtubule organiser (spindle pole body in *S. cereveisiae*). (B) The centromeres remain clustered and close to the nuclear envelope in the Rabl configuration. (C) During early meiosis the telomeres become attached via SUN/KASH proteins (not shown) to the nuclear envelope and then (D) cluster near the microtubule organising centre in the bouquet configuration.

It has been known for over a century that chromosomes can adopt a highly polar organisation in which centromeres are clustered with chromosome arms occupying different latitudes according to arm length (Rabl organisation [4]; Fig. 2B). The Rabl configuration is regularly seen in interphase cells of plants and other species, but it is not universal and is absent from most mammalian interphase cells (e.g. see [5], [6], [7], [8], [9], [10], [11], [12]). The Rabl organisation is a well established feature in *S. cerevisiae*, demonstrated by cytological, genetic and physical techniques [13], [14], [15], [16], [17]. In *S. cerevisiae* the Rabl configuration chromosome ends (telomeres) are dispersed but those from chromosomes of a similar size are more likely to be close to each other [17].

During the leptotene/zygotene transition of meiosis telomeres attach to the nuclear envelope (Fig. 2 C) and move to a telomere-clustered bouquet formation (Fig. 2 D; reviewed in [2], [13], [18], [19], [20], [21]). Most organisms studied have a demonstrable bouquet stage, but the degree of polarisation varies considerably (see [2], [18], [19], [22]). For example, *Schizosaccharomyces pombe* chromosomes are highly polarised, with all telomeres confined to a small part of the nuclear volume, where as telomeres in other organisms are restricted to a broader region of the nuclear membrane [22], [23]. Telomere clustering is generally tighter in organisms with a micro-tubule organising centre to which the telomeres are closely associated [2].

The significance of the Rabl and bouquet configurations is not fully understood. The Rabl organisation could be a relic of the preceding mitotic anaphase [6]. In *S. cerevisiae* meiosis it is possible that the Rabl configuration contributes to early centromere pairing [24]. In other organisms the structure is weak or absent in premeiotic cells [6], [25]. Over several years many genes have been identified in various organisms as being required for relocalisation of telomeres to the nuclear envelope (reviewed in [19], [26], [27]). Using *S. cerevisiae*, several laboratories have examined mutants in these genes to determine the significance of the Rabl to bouquet transition. Chromosome movement into the bouquet structure is brought about by telomere interaction, via adapter proteins, with the perinuclear cytoskeleton [28], [29], [30]. Location and movement of telomeres on the nuclear periphery requires a complicated and diverse array of functionally conserved proteins that interact directly with telomeres (e.g. Ndj1/Tam1 [31], [32], [33] and yKU70/80 [30], [34], [35]), link telomere bound proteins to the nuclear membrane (SUN domain proteins e.g. Mps3 [33], [36]) and link trans membrane proteins to the cytoskeleton (KASH or KASH-domain proteins e.g. Csm4 [37]).

The timing of meiotic chromosome organisation overlaps with that of recombination. The functional interrelationship between these two meiotic activities is not well understood, and while they influence each other they are not interdependent [33], [38], [39], [40]. For example, deletion of *S. cerevisiae NDJ1/TAM1* causes telomeres to become internalised and less mobile, with a change in crossover frequency and distribution, and delayed first meiotic division [31], [32], [33], [37], [41], [42]. Similarly deletion of yKu70 (*HDF1*) disrupts telomeres attachment and bouquet formation [35]. Chromosome pairing in such strains is delayed and the rate of segregation errors (non-disjunction) increases in some, though not all, reports [31], [32], [35], [37]. The major increase in nondisjunction in ndj1/tam1 mutants is for nonrecombinant chromosomes reliant the distributive segregation system [31], [32], [33]. These observations support the view that the bouquet contributes to chromosome pairing but it cannot be essential. While chromosome movement is probably universally important, the contribution of the bouquet varies widely between species. In some organisms chromosome pairing precedes bouquet formation [20], and in *S. pombe* the role of bouquet genes extends beyond pairing, to the proper regulation of the spindle pole body [43].

### Yeast chromosomes are highly motile at the onset of meiosis

Recent live cell studies have revealed that chromosome movement in budding yeast meiosis extends well beyond a simple movement of centromeres out of the Rabl configuration, and telomeres into the bouquet formation. From early prophase I, at least until synapsis is complete at pachytene, there are continuous rapid shifts in chromosome position. These can separate whole chromosomes from the main chromosome mass, causing shape changes in the nuclear membrane [33], [41], [44]. The movements in yeast are telomere led and unequal throughout the length of chromosomes, with the centromeres sometimes being appreciably less motile, and whole chromosomes transitioning from being rapid movers to stationary [33], [44]. This activity is dependent on actin, ATP and various proteins also needed for telomere location on the nuclear periphery and bouquet formation [29], [33], [41], [44]. Similarly, studies and modeling from plants have also shown that chromosome movements leading to telomere clustering require microtubules and are directional [45], [46], [47]. The chromosome movement is a highly organised and regulated feature of the meiotic programme, not Brownian motion. As prophase I in *S. cerevisiae* proceeds and chromosomes become more paired and then synapsed, the speed and tendency for movements reduces [33]. That chromosome movement is most vigorous in early prophase I has led to a widely held view that it stirs unpaired chromosomes, both to help homologues to find each other and to break up unwanted prexisting (ectopic) interactions [26], [33].

### Chromosome pairing is also important to avoid unwanted interactions

While chromosome pairing has the more obvious role of bringing homologues close enough to each other to recombine, it probably also has an important role in separating unrelated chromosomes or chromosome regions. All genomes contain a degree of DNA sequence repetition due to the presence of transposons, *pseudo* genes and gene families evolved from a single locus. Repeated sequences can be dispersed on many chromosomes, or they can be positioned at nonallelic loci on homologous chromosomes. Early in meiosis there is a chance that dispersed repeated sequences will make contact, and compete effectively with allelic sequences for pairing. When this happens there is an opportunity for so called ectopic recombination between nonallelic sequences. Ectopic recombination between diverged repeated sequences is largely repressed by chromatin structure and the mismatch repair system [48], [49], [50], but it occurs at measurable frequencies in various organisms, including budding yeast [51], [52], [53], [54], [55], [56], [57]. Avoiding ectopic crossovers is important because they alter chromosome structure creating translocations. These disrupt chromosome segregation and increase the risk of infertility or abnormal offspring (e.g. [53], [56], [57], [58]).

In *S. cerevisiae*, genetic experiments have used the efficiency of ectopic recombination events (as compared to allelic recombination) as a measure both homologue and heterologue chromosome juxtaposition [14], [58], [59], [60], [61]. Among conclusions drawn from these experiments is the notion that the chance of ectopic interactions is related to telomeres in two ways. Firstly, the distance of interacting loci from telomeres influences their chances of interaction [58]. Secondly, the location of telomeres on the nuclear periphery influences the efficiency of ectopic recombination [59], [60]. Thus, the telomere led movements that contribute to the bouquet structure and further pairing may be important for disruption of unwanted contacts between repeated sequences [26], [33], [59], [60].

### Modeling the influence of the Rabl and bouquet conformations on chromosome juxtaposition

One difficulty with genetic experiments is that pleiotropic effects are very difficult to separate from direct effects. For example, mutating a gene that modifies both chromosome movement and recombination makes it difficult to determine which of these aspects (either, or both) is directly responsible for an observed chromosome pairing defect. To augment what has been learnt from genetic and cell biology studies, we set out to develop an *in silico* test for the possible significance of chromosome tethering to the nuclear envelope, with or without clustering forces.

We developed polymer statistic models of chromosome behaviour that can incorporate a diverse range of centromere and telomere clustering, reminiscent of the Rabl and bouquet structures. The model has been used to investigate the potential roles of chromosome tethering and clustering forces upon the likelihood of loci becoming physically close to each other. While the modeling process can be used for any organism, we have set parameters to model the widely used experimental Eukaryotic microbe *S. cerevisiae*. Our main goal was to determine whether or not simply moving to the bouquet formation could increase the chances of close homologue juxtaposition, and reduce the chances of unwanted ectopic contacts. The model supports the view that telomere led movements into the bouquet structure can be an aid to chromosome pairing. We found that chromosome length and persistence length (chromosome flexibility) have a measurable difference on how beneficial the bouquet structure might be to chromosome pairing.

## Results/Discussion

We have developed a modeling process that can be summarised as follows. The software generates sample chromosome trajectories with defined contour length (i.e. short, medium or long chromosomes) and defined persistence length (i.e. flexible or rigid). The chromosomes are split into 300 equidistant notional genetic loci and are placed at random into a confined spherical space (the membrane bound nuclear volume). While *in vivo* chromosomes have positive volume that reduces the nuclear space available to be filled by another chromosome, we have not included this parameter in the current study (see Materials and Methods). A chance of turn/change in direction traveling along the chromosome is applied according to the persistence length. Further variables cause either the centromeres or telomeres to be tethered on the outer shell of the nucleus (nuclear membrane), and apply clustering forces to centromeres or telomeres. As defined here, clustering forces provide a greater than random chance that homologous centromeres or telomeres would be located close to each other.

Compared to available computer models, the ability to capture the effect of directional-biasing of centromere/telomeres represents a contribution towards more realistic modeling of chromosomes, and allows more accurate inference of important chromosome parameters. Most works assume an unconfined worm-like chain (WLC) model [26], [62] or else spherically confined but untethered WLCs [63]. Other models incorporate centromere or telomere tethering [64], but because they were developed to model interphase chromosomes they do not incorporate centromere/telomere clustering, a striking property of chromosome architecture *in vivo*.

We have used a variety of different parameters, informed by previous observations [46] (see Materials and Methods), to generate a time course of *S. cerevisiae* chromosome pairs moving from the Rabl configuration through four likely phases of bouquet development [46] (defined here as Telomeres Tethered without clustering and Early, Loose and Tight Bouquets). These are compared with a relatively unconstrained (untethered but spherically confined) condition, No Tether.

Because chromosome size likely influences nuclear distribution [65], the conditions were tested on three chromosomes of differing lengths. The shortest was modelled on yeast chromosome I (∼240 Kb), a middle sized chromosome nearly 4-times longer (such as chromosome XVI; ∼950 Kb) and a long chromosome more than 6-times longer than chromosome I (such as chromosome IV; ∼1530 Kb). At each step in the time course information is reported for 177,000 nuclei on the relative juxtaposition, pair wise, of the 300 notional loci on either homologous or heterologous chromosomes. The nuclear diameter was set at 2 µm based on a range of published estimates for haploid and diploid cells [66], [67].

In our first set of experiments we report the distances between nonallelic loci on a single chromosome. Next we report the distances between allelic loci on a pair of homologous chromosomes, this provides a view of the juxtaposition of a homologous pair. We have also considered two types of ectopic interactions. These provide the distances between nonallelic loci on homologous chromosomes and the distances between nonallelic loci on heterologous chromosomes. The computational and mathematical methods are described in Materials and Methods.

### Increasing persistence length decreases intrachromosomal contacts

**Figure 3:**
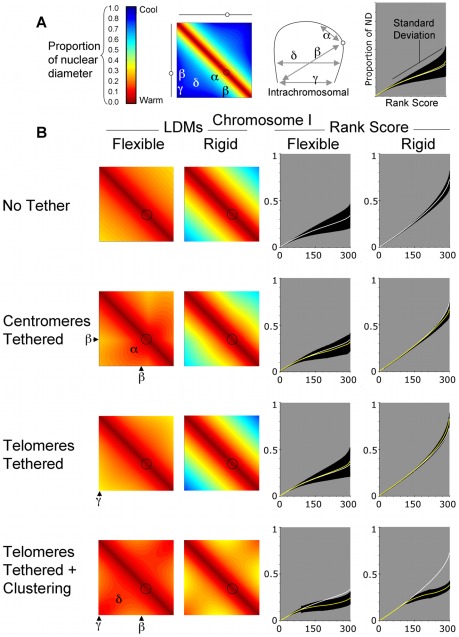
Intrachromosomal interactions are influenced by centromere and telomere location and persistence length – Chromosome I. (A) Output from the computer modeling (Materials and Methods) can be expressed in a heat map. The colours correspond to average distance between loci as a proportion of nuclear diameter. These are built up into locus distance maps (LDMs). The circles represent centromeres. The intersection between X- and Y-axes of the LDMs represents the distance between loci on the same chromosome. Different regions can be defined in the LDMs to examine how mean distances change between chromosome landmarks: such as between, (α) the centromere and interstitial region, (β) the centromere and a telomere (γ) opposite telomeres and (δ) interstitial regions on opposite arms. The graph provides a key for those displayed in (B). (B) LDMs are provided for a chromosome modelled on *S. cerevisiae* chromosome I (240 Kb) tested at two persistence lengths/rigidity and in four different configurations with respect to probable centromere and telomere location in the nucleus. In No Tether chromosome are located randomly in the nuclear volume, Centromere Tether means the centromere was located at the nuclear periphery, Telomeres Tethered means that both telomeres were tethered to the nuclear periphery at independent locations and Telomeres Tethered plus Clustering means there was an additional high chance of telomeres being located close to each. The two persistence lengths used to define flexibility were set at 0.2 µm and 2.0 µm and the nuclear diameter was set at 2 µm. Rank score graphs indicate the mean distances between loci collected into 300 bins after ranking. The data for No Tether is repeated in white lines for comparison to the yellow lines, which are the data for the other conditions. The black areas indicate standard deviation. Diagrams with showing the telomere distributions use a sample of 500 cells for each condition.

We first investigated how the different physical properties would affect the layout of individual chromosomes in the nucleus. For a particular choice of chromosome parameters (defining chromosome architecture), an average of all pair wise intra-chromosome distances could be calculated from sample trajectories to yield a matrix of average distances. This matrix is represented in a heat map, referred to here as the intrahomologue locus distance map (LDM; Fig. 3 A). The colour code chart indicates the relative distances as a proportion of nuclear diameter (ND). The gradation is from deep red (zero distance) through yellow and green to deep blue (maximum distance equal to the nuclear diameter; Fig. 3 A). By locating different loci on the horizontal and vertical axes, the colour at the intersection provides the mean distance between two loci. For example, the telomere-to-telomere distance is given by the region indicated γ in Fig. 3 A.

**Figure 4 pcbi-1002496-g004:**
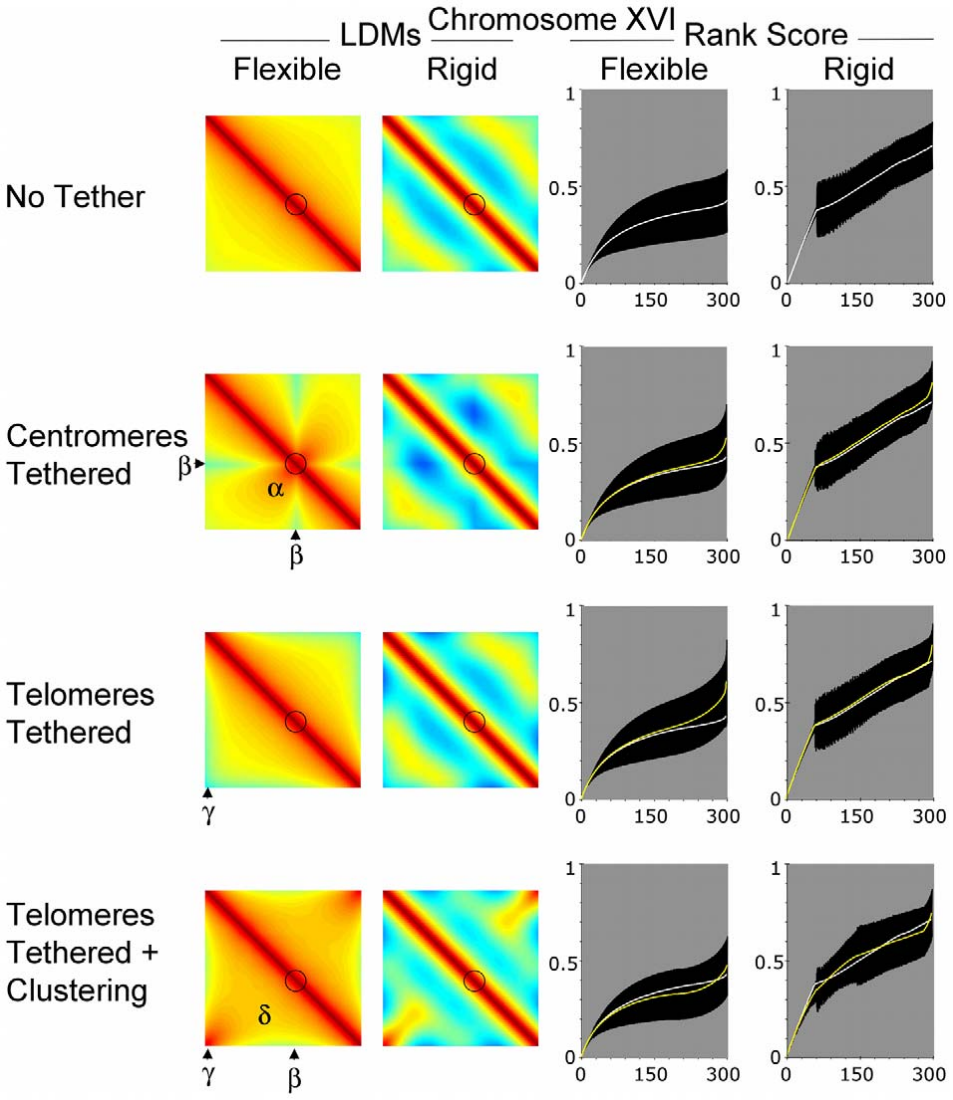
Intrachromosomal interactions are influenced by centromere and telomere location and persistence length – Chromosome XVI. Output from the computer modeling as described for Fig. 3 using a chromosome modelled on *S. cerevisiae* chromosome XVI (950 Kb). All conditions are as described for Fig. 3.

**Figure 5:**
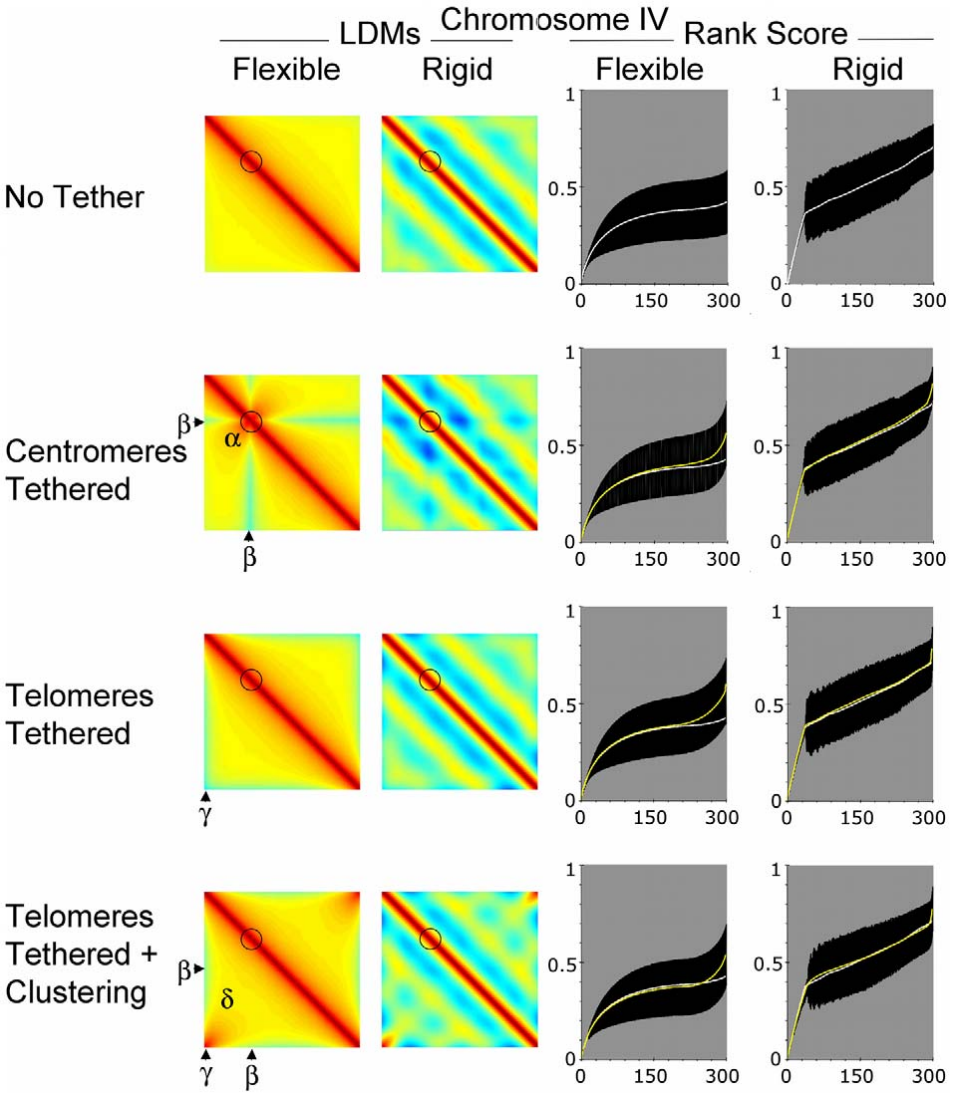
Intrachromosomal interactions are influenced by centromere and telomere location and persistence length – Chromosome IV. Output from the computer modeling as described for Fig. 3 using a chromosome modelled on *S. cerevisiae* chromosome IV (1530 Kb). All conditions are as described for Fig. 3.

The layout of each of the three chromosomes was tested under two flexibility regimes, as defined by the chromosomes' persistence length. The persistence lengths used were informed by previous measurements of *S. cerevisiae* chromosomes, with the most flexible value in line with interphase measurements, 0.2 µm [62] (but slightly larger than outside estimates from 3C modelling [68]). The more rigid case, 2.0 µm, is between the flexible values and high values inferred from pachytene chromosomes [26]. Results from both persistence lengths are displayed in Figs. 3 B, 4 and 5 for short, medium and long chromosomes, respectively. We also used a range of position restricting conditions. These were, No Tether (spherically confined only), Centromeres Tethered to the nuclear envelope, Telomeres Tethered to the nuclear envelope and Telomeres Tethered plus Clustering forces.

For all chromosomes tested, and in all four restraining conditions, increasing the persistence length (and therefore rigidity) had the expected effect of increasing the mean distance between loci. Thus, the intrachromosomal LDMs for rigid chromosomes in Figs. 3 to [Fig pcbi-1002496-g004] 5 are cooler than for flexible chromosomes. Another way to view the data is by rank scoring the distances. For comparison later with the 300 interallelic rank scores, the 90,000 mean distances (of 177,000 samples) per chromosome were ranked and then binned into 300 mean scores. Comparing the flexible and rigid rank score graphs (Figs. 3 to [Fig pcbi-1002496-g004] 5) shows that a wider range of mean distances is adopted when the chromosomes are more rigid.

### Restricting the position of centromeres or telomeres of flexible chromosomes modified the distribution and range of intrachromosomal spread

For flexible chromosomes, the centromere tether changed the distribution of distances for each chromosome. Pericentromeric regions were more likely to be close to each other, thus on the intrachromosomal LDMs there is more red/orange around the centromeres (Figs. 3 to [Fig pcbi-1002496-g004] 5; Centromeres Tethered region α). This change was accompanied by an increase in mean distances between the centromeres and distant loci, causing the intrachromosomal LDMs to become more yellow/green in regions indicated β. The rank scores indicated that centromere tethering caused a reduction in the range of mean distances for the flexible chromosome I (Fig. 3; Centromeres Tethered), but an increase for chromosomes XVI and IV (Figs. 4 and 5, Centromeres Tethered). While by eye these differences may seem small and to affect a small proportion of the chromosomes' length, it is noteworthy that the mean curves for No Tether and Centromeres Tethered are statistically significantly different from each other (p<0.01, using both a Kolmogorov-Smirnov and Wilcoxon rank-sum test to compare the distribution of the means for all 90,000 intrachromosomal distances, where each mean is averaged across the population of ∼177,000 cells for each chromosome and condition). Thus, the overall impact on tethering is chromosome size dependent.

An influence from chromosome size is also apparent when looking at the data for Telomeres Tethered without clustering force. When telomeres of the same chromosome are randomly attached to the nuclear periphery they become relatively dispersed compared to when they were free to lie anywhere with the nucleus. This is seen in region γ of the intrachromosomal LDMs, which become cooler when compared to No Tether (Figs. 3 to [Fig pcbi-1002496-g004] 5). For all flexible chromosomes this causes a widening of the range of mean intrachromosomal distances that is statistically significant (p<0.01, using Kolmogorov-Smirnov and Wilcoxon rank-sum tests). The longer chromosomes are more spread out in the condition Telomeres Tethered than chromosome I, widening the mean gap further, presumably because the telomeres can be located at more distant sites on the nuclear membrane (Figs. 3 to [Fig pcbi-1002496-g004] 5; Telomeres Tethered, compare rank scores).

These observations are consistent with those seen in previous polymer-statistics models, in which tethering of centromeres or telomeres to the nuclear periphery increased the average distance between opposite telomeres [64].

### Intrachromosomal spread for small chromosomes is decreased by clustering opposite telomeres of the same flexible chromosome on the nuclear periphery

We also tested the effect of tethering telomeres to the nuclear periphery with a strong chance of being located close to each other, as would be seen in a bouquet structure (ν = 50, see Materials and Methods; Figs. 3 to [Fig pcbi-1002496-g004] 5, Telomeres Tethered LDMs are warm in region γ). For chromosome I the clustering of telomeres caused a significant reduction in mean distances for a large proportion of loci (Fig. 3; Telomeres Tethered plus Clustering, rank scores). By effectively pulling the short chromosome into a U-shape, interstitial regions on opposite arms become closer than in any other condition tested. This is shown by the deepening red in region δ, and on the rank score graph the distribution of means is lower than that in other conditions. The effect is similar but less pronounced on chromosome XVI in region δ (Fig. 4 Telomeres Tethered plus Clustering) as more loci will be further away from the joint tether site. Also important is the observation that the mean distances increased in region β compared to untethered chromosomes. For chromosome IV the increase in distance in the region β was sufficient to increase the overall range of mean distances compared to the No Tether condition (Fig. 5; Telomeres Tethered plus Clustering).

At distance from the tether, order imposed by clustering gives way to changes in chromosome trajectory and the effect of the clustering on the mean distance between loci wanes. This observation has implications, predicting that any influence of the bouquet on chromosome pairing will be chromosome length limited.

### Rigid chromosomes follow the same trends as flexible chromosomes but boundary effects are more likely to influence trajectory

The different conditions effected rigid chromosomes in similar ways to flexible chromosomes, particularly for the small chromosome I (Figs. 3 to [Fig pcbi-1002496-g004] 5). For the two longer chromosomes the combination of rigidity (fewer turns in trajectory) and length means that collision with and deflection from the boundary is more likely (see e.g. [69]). The deflection of chromosome ends away from the nuclear periphery boundary can increase the chances of loci on distant chromosome regions coming close to each other in the nuclear volume. On the intrachromosomal LDMs this caused a striated pattern of alternating warmer and cooler colours (Figs. 4 and 5; rigid LDMs). This effect also reduces or reverses the impact of tethering and clustering of either centromeres or telomeres (Figs. 4 and 5; compare rank scores flexible versus rigid).

### Homologue juxtaposition is not influenced by chromosome length when there is no tethering to the nuclear periphery, but increasing rigidity increases the distance between alleles

**Figure 6:**
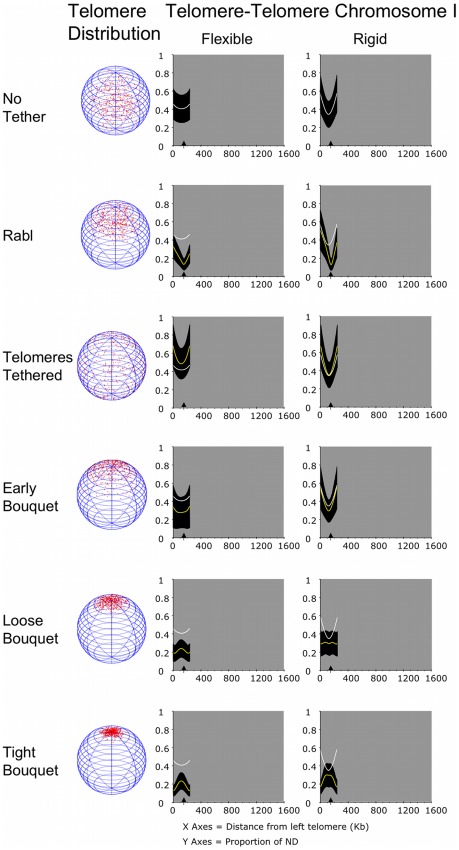
Clustering forces could play a major role in the pairing of small chromosomes. The output of distances between alleles on a pair of chromosomes modelled on chromosome I of *S. cerevisiae*. The white and yellow lines are mean distances (from 177,000 randomly selected trajectories) plotted from one end of the chromosome between 300 equidistant notional allelic loci, expressed as a proportion of the nuclear diameter. The black area denotes standard deviation. The arrowhead on the X-axis indicates the position of the centromere. The mean distances for No Tether have been included on other graphs in white for comparison. For each chromosome layout used samples of telomere distributions (from 500 nuclei using the shorter persistence length) are indicated by the red dots in the nuclear spheres to the left. Diagrams showing telomere distributions use a sample of 500 cells for each conditions. A sample of chromosome I of trajectories, including a wider range of Rabl conditions, is provided in Figs.s S1, S2 and S7.

We set out to determine how similar parameters would impact on the proximity of homologous chromosomes in an otherwise empty nucleus. For each condition we measured the proximity of 300 allelic loci along homologous chromosome pairs I, XVI and IV. A sample distribution of telomeres is indicated for each flexible chromosome during the Rabl to Tight Bouquet time course (Figs. 6 to [Fig pcbi-1002496-g007] 8).

**Figure 7 pcbi-1002496-g007:**
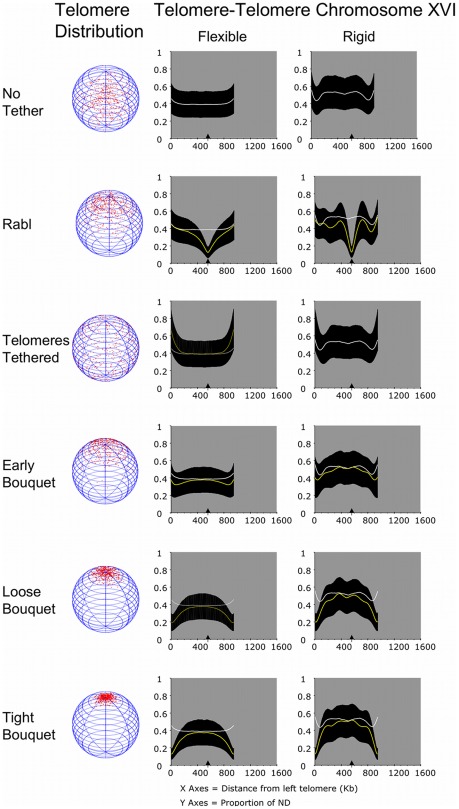
The influence of clustering forces over interhomologue distance for larger chromosomes is limited by distance from the tether site, and dependent on chromosome rigidity. The output of distances between alleles on a pair of chromosomes modelled on chromosome XVIs of *S. cerevisiae*. All aspects of the layout are as for Fig. 6. A sample of chromosome XVI of trajectories, including a wider range of Rabl conditions, is provided in Figs.s S3, S4 and S7.

**Figure 8:**
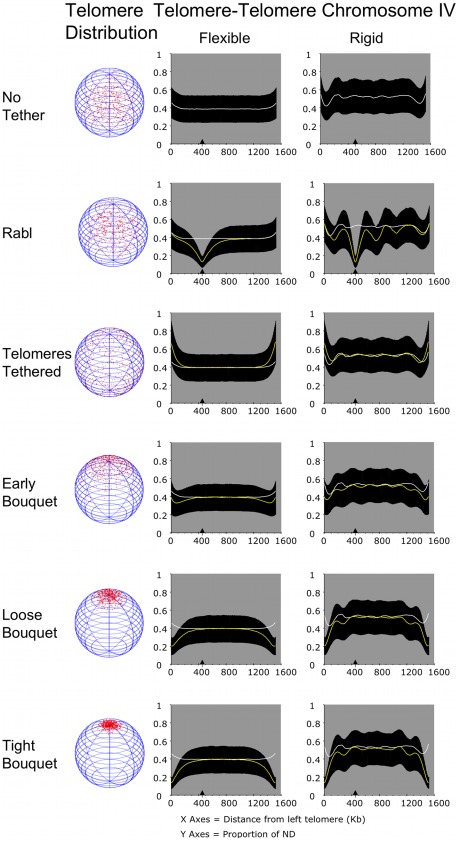
Increasing chromosome size further reduces the influence of clustering forces and increases the significance of chromosome rigidity on distance between homologues. The output of distances between alleles on a pair of chromosomes modelled on chromosome IV of *S. cerevisiae*. All aspects of the layout are as for Fig. 4. A sample of chromosome IV of trajectories, including a wider range of Rabl conditions, is provided in Figs.s S5, S6 and S7.

In the first condition no special constraints were given to the locations of centromeres or telomeres, this provided a baseline of interhomologue juxtapostion to compare with more restrained conditions (Figs. 6 to [Fig pcbi-1002496-g007] 8; No Tether). Even though there is a 6-fold difference in length between chromosomes I and IV, for all three flexible chromosomes the distance between alleles on homologous chromosomes ranged only from approximately 0.40 to 0.45-times ND.

By definition, rigid chromosomes are less likely to make a turn in direction, and therefore they are more spread out as indicated by the measurements of intrachromosomal distances. This has an impact on the position of chromosomes in the nucleus and, therefore, homologue juxtaposition. Increasing rigidity causes an increase in the mean distances between alleles compared to the flexible chromosomes (Figs. 6 to [Fig pcbi-1002496-g007] 8; No Tether, compare Rigid with Flexible). It is noteworthy that as chromosome size increases, there is a fluctuation in pairing contacts due to boundary effects (Figs. 7 and 8; No Tether, Rigid).

Next, five stationary conditions were used to mimic time course sampling of a continuous process with chromosomes moving from a Rabl configuration to a tight bouquet formation. The Rabl configuration was created by localising centromeres to the nuclear periphery with strong clustering forces (ν = 50; see Materials and Methods). For Telomeres Tethered, telomeres were tethered to random sites on the nuclear periphery. We then utilised three levels of increasing clustering tendencies for tethered telomeres. These are referred to as Early Bouquet, Loose Bouquet and Tight Bouquet. Respectively, the three bouquets had v values of 5, 10 and 50 (Materials and Methods).

### Homologues are closer together in the Rabl configuration compared to untethered, but the influence of clustering forces gradually wanes with distance from the centromere

For the flexible chromosomes, when centromeres are in the Rabl configuration alleles closest to the centromere were separated by less than 0.1-times ND (Figs. 6 to [Fig pcbi-1002496-g007] 8; Rabl, Flexible). This compares with a separation of ∼0.40-times ND for pericentromeric alleles in the No Tether condition (Figs. 6 to [Fig pcbi-1002496-g007] 8; No Tether, Flexible). Moving away from the clustered centromeres leads to a gradual increase in the mean distance between alleles (Figs. 6 to [Fig pcbi-1002496-g007] 8; Rabl Telomere-Telomere graphs, yellow lines tend towards white line moving away from the centromere). Chromosome I is not long enough for the influence of the Rabl configuration to completely wane near the telomeres. For chromosomes XVI and IV the mean distance between alleles converges with that for untethered chromosomes. Thus, as chromosomes become longer a decreasing proportion of the total length of homologues will be influenced by the Rabl configuration.

Increasing chromosome rigidity had the effect of causing a more rapid drop off of the clustering influence for all chromosomes (Figs. 6 to [Fig pcbi-1002496-g007] 8; Rabl, compare Flexible and Rigid). This is shown for chromosome I by the near convergence of the data for Rabl with the data for No Tether on the left arm furthest from the centromere. However, mean interhomologue measurements are still closer to each other than for No Tether (in all cases, p<0.01, using Kolmogorov-Smirnov and Wilcoxon rank-sum tests).

The same trends are apparent for the longer chromosomes, but there is some periodicity to the homologue juxtaposition due to boundary effects (Figs. 4 and 5; Rabl, rigid).

### Tethering telomeres without clustering reduces the chances of homologues being close to each other

For all three chromosomes used, tethering telomeres tended to reduce the chance of interhomologue proximity compared to the No Tether condition (Figs. 6 to [Fig pcbi-1002496-g007] 8, Telomeres Tethered). This is most apparent at the tether sites, as without clustering forces tethered homologous telomeres could be constrained to distant sites on the nuclear envelope. At distance from the tethered telomeres the proximity of homologues tends towards that seen for untethered chromosomes.

The shortest chromosome is not long enough for any loci to escape the relative disruption to homologue juxtaposition created by Telomeres Tethered without clustering forces. Therefore, in this condition pairing short homologues might be more difficult than pairing long chromosomes, which for a portion of their length are as close as in the No Tether condition.

Overall, the mean distance between alleles is increased by loss of the Rabl configuration. Thus, our model could explain why just prior to meiosis, yeast chromosomes appear to be paired and this paring is lost on entry into meiosis until meiotic chromosome pairing is established [70], [71], [72].

### Increasing clustering forces at the telomeres incrementally improves the juxtapositioning of short homologues

Early Bouquet formation was modelled by creating a small chance of telomere clustering (ν = 5). For flexible short chromosomes in Early Bouquet, the range of distances between alleles was 0.27- to 0.34-times ND (Fig. 6; Early Bouquet, Flexible). This compares to a range of 0.40- to 0.45-times ND for No Tether and a range of 0.47- to 0.67-times ND for Tethered Telomeres without clustering. Thus, a relatively small chance of telomeres being close to each other creates a measurable improvement in homologue juxtaposition over many Kb.

Increasing the clustering forces to create the Loose and Tight Bouquets brought telomeres even closer together (respectively, to within 0.20- and 0.12-times ND). As seen for the Rabl configuration, the influence of these clustering forces reduced moving away from the cluster site. This caused a convergence towards the mean distances between homologues established for the No Tether condition. However, chromosome I is sufficiently short that even at its mid point (where it bows towards the central nuclear volume ∼120 Kb from each telomere), the chance of close juxtaposition is higher compared to chromosomes with No Tether and Telomeres Tethered (Fig. 6).

### Longer homologous chromosomes benefit from bouquet formation over a shorter proportion of their length

For flexible chromosome XVI the tight bouquet also brought the entire length of the chromosome pair into closer proximity. At the mid point of the chromosomes, the distance between alleles was ∼0.37 ND, where it converged on the distances recorded for No Tether and Telomeres Tethered with no clustering (Fig. 7).

The longer chromosome IV pair gained close juxtaposition over a similar length to the chromosome XVI pair. Thus, up to ∼400 Kb from each telomere the distance between alleles was closer than for the same chromosome with No Tether (Fig. 8; Loose and Tight Bouquet, Flexible compare yellow and white lines on graphs).

While we do not know what would be a critical distance between alleles on homologues to define them as paired or not in meiosis, the implication is that longer chromosomes as a whole might benefit less from the bouquet formation than short chromosomes. Although as shown below, the measure of benefit *in vivo* would be dependent on the true persistence length of chromosomes.

### Chromosome pairs that are rigid are further apart than flexible chromosome pairs, but long chromosomes may benefit from periodicity

Our modeling of intrachromosomal contacts illustrates the importance of chromosome rigidity in defining trajectory through the nuclear volume. In meiosis it is thought that chromatin cycles through rounds of expansion and contraction, which presumably change their flexibility or contour length [3]. Such oscillatory changes could have an impact on chromosome pairing. Here we have considered two stable states of chromosome rigidity and analysed their impact on homologue juxtaposition.

**Figure 9:**
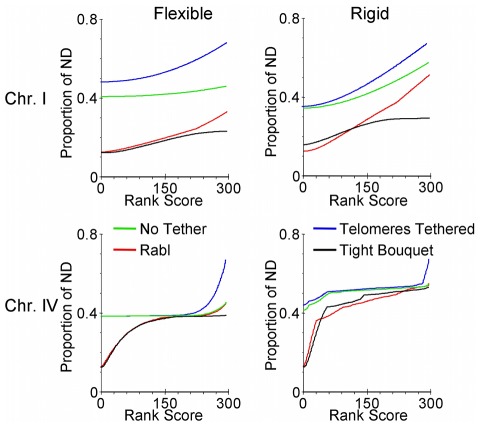
The mean distances between alleles are shorter when homologues are in a tight bouquet compared to non bouquet configurations. Comparison of rank scores for 300 mean distances between alleles on homologues with No Tether, Telomeres Tethered or in either Rabl or Tight Bouquet with flexible or rigid chromosomes. X-axis is rank score, Y-axis is mean distance as a proportion of nuclear diameter.

We found that making the chromosomes more rigid by increasing their persistence length caused an increase in the average distance between loci for all tethered chromosomes. The impact of increasing rigidity was more modest on the small chromosome I pair than the largest chromosome IV. Considering the Tight Bouquet, for chromosome I the rigid condition increased the range of separation between alleles from 0.12- to 0.23-times ND for the flexible chromosomes to 0.16- to 0.29-times ND (Fig. 9; Tight Bouquet compare rank scores, chromosome I flexible and rigid). For the chromosome IV pair the range of distances between alleles increased from 0.12- to 0.38-times ND for the flexible chromosome to 0.12- to 0.52-times ND for the rigid chromosome (Fig. 9; Tight Bouquet compare rank scores, chromosome IV flexible and rigid). Even thought the mean distances for the rigid chromosome IV are wider than for the flexible chromosome IV, they are significantly lower than in the No Tether condition and Telomeres Tethered without clustering (Fig. 9; p<0.01, both a Kolmogorov-Smirnov and Wilcoxon rank-sum test).

A potential benefit to pairing long chromosomes is the distribution of closer juxtaposition periodically along the length of the chromosomes (Figs. 7 and 8). We suggest this is due to boundary effects created by the combination of rigidity and length. Such periodicity could help pairing at distance from the tethered telomeres. This point illustrates the importance in accurate information about chromosome persistence length as it influences chromosome trajectory and the potential impact of the bouquet structure.

### The bouquet improves homologue juxtaposition compared to the Rabl configuration

The clustering forces we have used for centromeres in the Rabl configuration and telomeres in Tight Bouquet were equal. As modelled so far both the Rabl and Tight Bouquet configurations increase homologue juxtaposition relative to the hypothetical state of No Tether, and the Tethered Telomeres with no clustering forces (Figs. 6 to [Fig pcbi-1002496-g007] 8). On average, however, the bouquet can reduce further the overall distribution of mean distances between alleles (Fig. 9).

We do not know if *in vivo* the degree of clustering at centromeres in Rabl or telomeres in bouquet are similar or significantly different. If in *vivo* centromeres are less tightly clustered in Rabl than telomeres in the bouquet, then the bouquet would produce even more advantage to chromosome pairing than this model suggests. On the other hand, if clustering *in vivo* is tighter at centromeres and this is not lost in movement to the bouquet then the bouquet may be more dispensable. It will be interesting to determine the *in vivo* relative clustering tendencies in these two polarised arrangements for a range of organisms.

### Mean distances between nonallelic loci on homologues are greater than those between alleles in the Tight Bouquet condition

All eukaryotic genomes contain a degree of repetition of genomic DNA sequence and this is a potential source of problems during meiosis. Genetic studies show that chromosome pairing has the unwanted effect of increasing interhomologue ectopic contacts [58], [59], [60]. It therefore makes sense that there should be a counter pairing process to discourage ectopic interactions.

**Figure 10:**
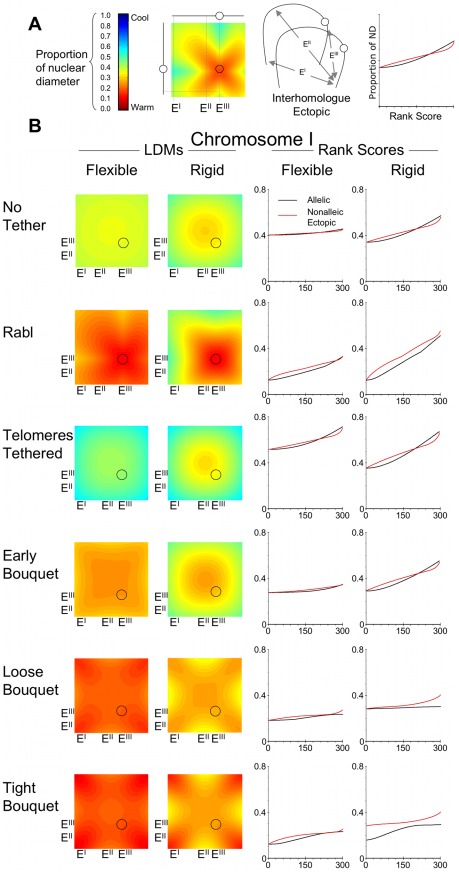
The mean distances between nonallelic loci on homologous chromosomes increases with the Tight Bouquet and increasing chromosome rigidity. The output of distances between alleles on a pair of chromosomes modelled on chromosome I of *S. cerevisiae* expressed in (A) interhomologue LDMs, the colour coding indicates distances between loci on homologous chromosomes, expressed as a proportion of nuclear diameter. The two chromosomes on the X- and Y-axis of the LDMs are homologous partners. X- and Y-axis intersections on the interhomologue LDMs represent distances between alleles (on the diagonal), and distances between nonallelic loci, which are off diagonal. Examples of such ectopic interactions are shown as areas on the LDMs adjacent to E^I^, E^II^, E^III^. The circle in the LDM represents the position of the centromere. The graph indicates the use of the axes for those displayed in (B). (B) The interhomologue LDMs are organised with the same conditions described in Fig. 6. The accompanying graphs reveal on the rank scores of the 300 mean allelic distances and the 89,700 mean ectopic distances collected into 299 bins (each bin containing the average of 300 ranked mean distances).

**Figure 11:**
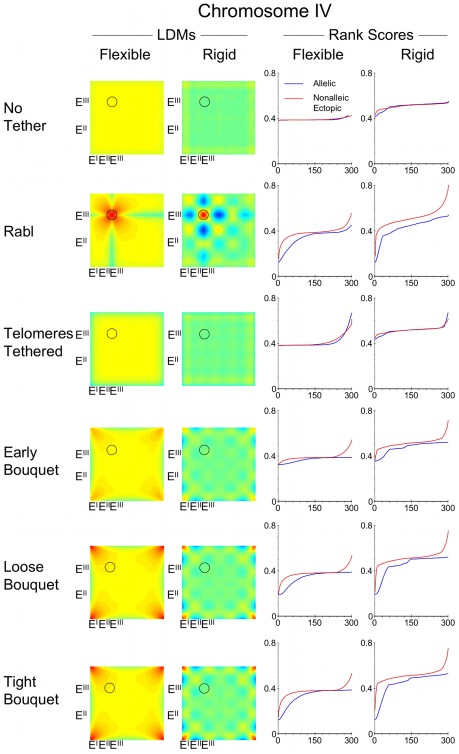
The main benefit of the Tight Bouquet for chromosome IV may be the increased distances between nonallelic loci. The output of distances between alleles on a pair of chromosomes modelled on chromosome IV of *S. cerevisiae* expressed in interhomologue LDMs and rank score graphs as described for Fig. 10.

We have illustrated the distances between nominal ectopic loci on chromosome I and IV homologues by plotting interhomologue LDMs (Figs. 10 and 11). The colour code chart indicates the relative distances between interhomologue sites as a proportion of nuclear diameter (Fig. 10A). The diagonal on the LDMs represents distances between alleles, with all off diagonal colour representing ectopic distance between nonallelic loci. The mean distances between alleles were rank scored for comparison with the mean ectopic distances, which were rank scored and then grouped into 300 bins. A few landmark examples of LDM areas representing potential interhomologue ectopic interactions are indicated (E^I^, E^II^, E^III^).

In the various conditions used, and for both flexible chromosomes I and IV, the trends in change in proximity of nonallelic loci on homologues mirrors that seen for allelic loci (Figs. 10 B and 11). In the condition of No Tether the colour range in the interhomologue LDMs along the diagonal is similar to that off diagonal. Viewing the mean distances as rank scores shows a high degree of overlap for allelic and ectopic distances.

The Rabl configuration causes the homologous chromosomes to be more aligned increasing the register between allelic loci. Thus the off diagonal colours on the interhomologue LDMs are on average cooler than on diagonal. As the centromeres have a strong tendency to be anchored and clustered, the furthest distances are between the centromere and the long arm telomere (Figs. 10 B and 11; Rabl, Flexible, E^III^ left border on LDM). The associated rank score graphs reveal the overall wider mean distances between ectopic loci compared to the mean distance between allelic loci.

In the condition of Telomeres Tethered all four telomeres can be widely separated on the nuclear envelope, and therefore there is little linear register between homologues. This causes the mean distances between ectopic loci and allelic loci to be more similar than in the Rabl configuration. As the bouquet becomes progressively tighter, the difference between mean ectopic and mean allelic distances increases. While by definition telomeres are close in Tight Bouquet (Figs. 10 B and 11; LDMs for Tight Bouquet are warm at position E^I^), other ectopic regions are cooler than the diagonal (regions E^II^ and E^III^). The rank score graphs for Tight Bouquet indicate that interhomologue ectopic mean distances are overall wider than allelic mean distances. As chromosome IV is longer than chromosome I, the potential gap between nonallelic loci on chromosome IV homologues is wider. As the telomeres are fixed on the nuclear periphery, it is not surprising that the greatest separation occurs between telomeres and interstitial regions (Fig. 10 B and 11; Tight Bouquet region E^II^). This is consistent with genetic data from yeast that indicates interhomologue ectopic recombination between telomeres is more likely than ectopic recombination between telomeres and distant interstitial loci [58], [59].

### Interhomologue ectopic distances are wider when chromosomes are rigid

When the chromosomes are more rigid both allelic and ectopic mean distances increase compared to flexible chromosomes. This is demonstrated by the general change to cooler colours in the rigid interhomologue LDMs (Figs. 10 B and 11). The increase in distance associated with making chromosomes more rigid is greater for nonalleic loci, thus in Tight Bouquet (rigid) there is more yellow/blue in regions E^II^ and E^III^. For chromosome I the maximum of mean distances between allelic sites increased from 0.23-times ND for Flexible to 0.29-times ND for Rigid. The maximum of mean distances between nonallelic sites increased from 0.25-times ND for Flexible to 0.38-times ND for Rigid. This trend is clearly demonstrated by the rank score graphs in which the gap between allelic and ectopic scores is wider for rigid chromosomes.

This phenomenon is also more exaggerated for the larger chromosome IV. The maximum of mean distances between allelic sites increased from 0.38-times ND for Flexible to 0.52-times ND for Rigid. The maximum of mean distances between nonallelic sites increased from 0.52-times ND for Flexible to 0.74-times ND for Rigid.

Thus while the improvement in close homologue juxtaposition caused by the bouquet is less for the longest versus the shortest chromosome, the longest chromosome may benefit more from the wider differential between interallelic and ectopic distances, particularly when rigid.

We next tested the degree to which chromosome tethering and the tendency for clustering forces impacts on the competition between allelic and ectopic interactions between heterologous chromosomes.

### Clustering forces and increasing chromosome rigidity create a chromosome size dependent bias for interhomologue juxtaposition over interheterologue juxtaposition

Related dispersed sequences among heterologous chromosomes have the potential to compete for chromosome interactions, which should be limited to between alleles. Avoidance of physical proximity between heterologues at the pairing stage would contribute to reducing the risk of deleterious interheterologue ectopic recombination. The highly polarised bouquet and rapid telomere led chromosome movement of *S. pombe* chromosomes have long been proposed as a size sorting mechanism [73]. Our *in silico* model supports the view that the bouquet acts as a size sorter.

**Figure 12:**
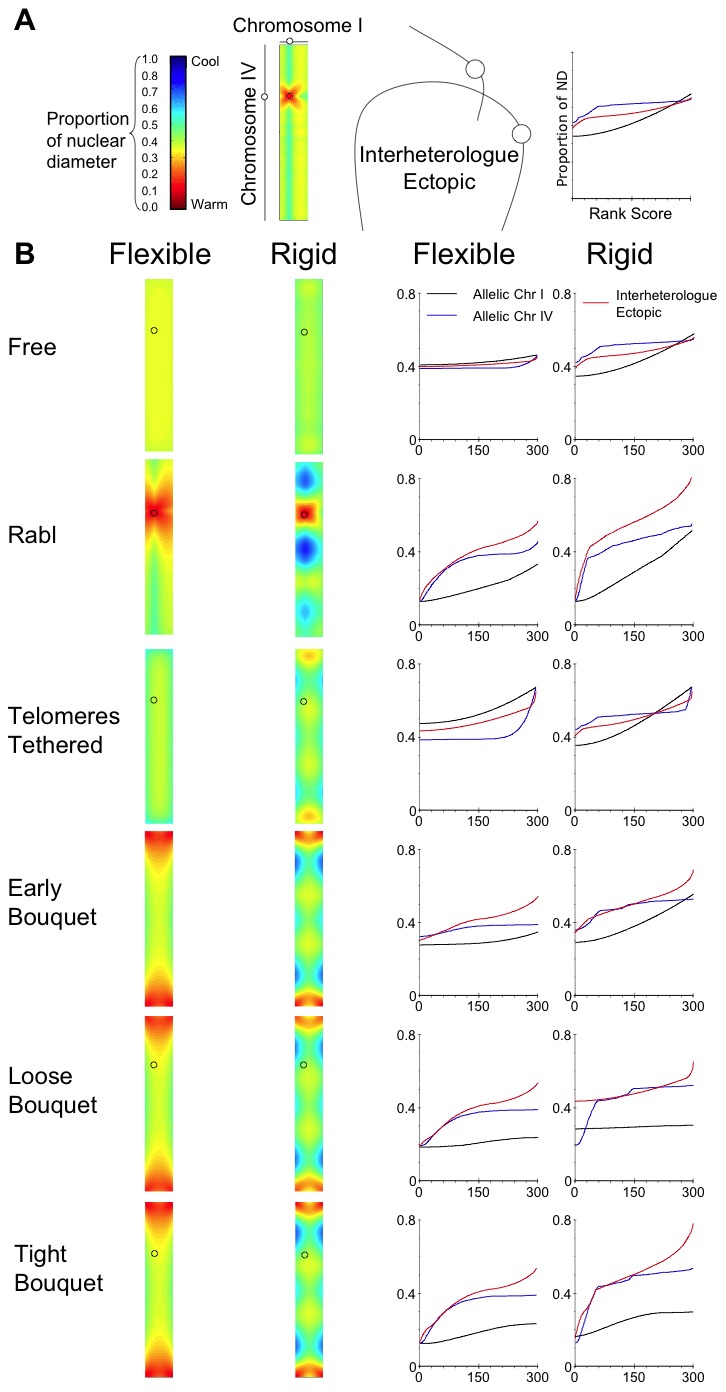
Ectopic interactions between heterologous chromosomes are reduced by chromosome tethering, clustering and rigidity. The output of distances between alleles on a heterologous pair of chromosomes modelled on chromosome I and IV of *S. cerevisiae* expressed in (A) interheterologue LDMs, the colour coding indicates ectopic distances between loci on heterologous chromosomes, expressed as a proportion of nuclear diameter. The two chromosomes on the X- and Y-axis of the LDMs are proportional to the heterologous chromosome, and each contains 300 equidistant notional loci. The graph indicates the use of the axes for those displayed in (B). (B) The interheterologue LDMs are organised with the same conditions described in Fig. 6. The accompanying graphs reveal the 90,000 mean ectopic distances, which were ranked and collected into 300 bins (each bin containing the average of 300 ranked mean distances). Allelic rank scores from data in Figs.s 9 and 10 are provided for comparison.

We measured the pair wise distances between all 300 notional loci on each of our shortest and longest chromosomes (i.e. 90,000 measurements). The distances have been plotted in interheterologue LDMs in Fig. 12, for the shortest (flexible) and longest (rigid) persistence lengths used.

For the flexible chromosomes in the condition of No Tether, the mean distances between heterologues are very similar to that between homologues (Figs. 12 B; No Tether, compare ectopic and allelic rank scores). As the Rabl configuration increases the chances of all centromeres being close to each other, pericentric regions of heterologous chromosomes I and IV are more likely to be close in the conditions that model Rabl (Fig. 12 B; Rabl, LDM is warmer around the centromeres). With increasing distance from the centromeres the distance between chromosomes I and IV increases more than the interallelic distances (Figs. 12 B; Rabl, LDM is cooler moving away from the centromeres; in rank score graphs the distances are higher for interheterologue ectopic).

This Rabl induced size sorting becomes lost in the Telomeres Tethered condition. Heterologues are further apart in Telomere Tethered than in No Tether (Fig. 12 B; Telomere Tether LDM is cooler than No Tether LDM), but the mean distances between chromosomes I and IV are intermediate between the mean distances between homologues (Fig. 12 B; rank score graphs). This supports the view that only tethering telomeres to the nuclear periphery might hinder the requirement to bias the proximity of homologues over the proximity of heterologues.

Introducing an increased chance for telomeres to be close to each other as the bouquet develops re-establishes size sorting. In the Tight Bouquet heterologous telomeres will by definition have a tendency to be close to each other (Fig. 12 B; Early to Tight Bouquet, LDMs are warmer near telomeres). Moving away from telomeres the distance between heterologues increases more rapidly than the mean distances between homologues, producing a greater separation (Fig. 12 B; Early to Tight Bouquet, compare rank scores). Chromosome flexibility influences the degree to which the gap increases between heterologues, compared to the mean distances between homologues. In the Tight Bouquet, for flexible chromosomes the maximum distance between chromosome IV homologues is 0.39- compared to 0.53-times ND between heterologues. But, with rigid chromosomes the maximum for chromosome IV homologues is 0.53-times ND compared to 0.77-times ND for heterologues. This suggests that when the chromosomes are rigid the bouquet may be less effective at bringing longer chromosomes into juxtaposition, but it is more effective at separating them from short chromosomes.

Taken together, the observations on large chromosomes suggest that for them the bouquet may be as important for disrupting unwanted ectopic interactions as fostering allelic interactions, particularly if they are relatively rigid. Chromosome flexibility is thought to fluctuate during prophase I, due to changes in chromatin compaction [3]. In particular, it is suggested that chromosomes would be more rigid during Leptotene than Zygotene [3]. If correct, this change in persistence length around the time of bouquet formation may be important to alternately separate heterologues and juxtapose homologues. The idea that the bouquet discourages interaction between heterologues is supported by genetic experiments in mutants unable to form the bouquet, as they show a 2-fold increase in ectopic recombination [59], [60].

### Concluding remarks

The model presented here argues that the attachment of telomeres to the nuclear envelope and a tendency to cluster them (forming bouquet structure) increases the chances of homologous chromosomes lying close to each other. The influence and potential contribution of the bouquet appears to be different for short versus long chromosomes. We suggest the bouquet could be a major contributing factor and possibly sufficient for pairing small chromosomes. Another important physical attribute of chromosomes that could influence their juxtaposition is rigidity. The model suggests that increasing rigidity has more of an effect on large chromosomes possibly helping to separate them from unwanted ectopic interactions. Importantly, increasing rigidity also reduces the chances of interheterologue ectopic interactions.

The differential importance of the bouquet structure to short and long chromosomes is consistent with modeling of yeast interphase chromosomes, which showed chromosome length influences positioning in the nucleus [65]. This difference may explain why on the one hand the bouquet is important and well conserved, while not being absolutely essential to chromosome pairing. Another issue worth considering is that telomere attachment to the nuclear envelope may have a function independent of chromosome pairing. As telomere attachment reduces homologue juxtaposition, the movements and bouquet structure may be there to counter this effect.

This model is the first one we are aware of that uses both telomere or centromere tethering to the nuclear periphery combined with a directional force applied to the tethered site, representing the effect of a microfilament network. While this work represents a significant improvement over current models available, it nonetheless has some notable limitations. In particular we have not considered excluded-volume interactions arising from other chromosomes or subnuclear structures such as the nucleolus [17], [65], [74]. Accounting for such excluded-volumes represents an obvious extension to this work, in particular by jointly modeling the entire genome (see e.g. [63]). Additional extensions to the model could be to allow simultaneous tethering and clustering of centromeres and telomeres, as the Rabl configuration may not be entirely lost when the bouquet forms [24], [65]. It will also be important to incorporate the rapid prophase chromosome movements [29], [33], [41], [44], the function of which is probably not restricted to bringing about bouquet formation. Introducing homology comparisons to bias homologue interactions will also be important to creating a more accurate model [46]. Including all of these additional factors however, will require a very significant increase in computer processing power.

Further measurements to define better the nuclear and chromosome size changes that take place in meiosis are important to inform the modeling process. For example persistence length measurements vary considerably in the literature, and probably along the chromosome length [44], [62], [68].

There is some evidence in genetic data to suggest short chromosomes are more susceptible than long chromosomes to nondisjunction in mutants lacking telomere tethering [37]. We are keen to test more directly, the prediction that small chromosomes are more susceptible to loss of the bouquet than large chromosomes.

With further refinements of the model we hope to determine if the physical constraints on chromosomes during pairing impact on which loci are more likely to recombine, and perhaps influence the genetic map and therefore evolution.

## Materials and Methods

### Polymer statistics for meiotic chromosomes

The behaviour of chromosomes has previously been investigated in terms of flexible or semiflexible polymers [75], [76], [77]. Unconfined worm-like chain (WLC) statistics, wherein the chromosome is modelled as a continuous polymer with parameterised stiffness, can also be used to model the statistical behaviour of chromosomes far from physical boundaries. Since unconfined WLC models admit closed-form solutions to many statistical measures of interest, including the expected distance between any two loci [78], [79], [80], [81], [82], the WLC has become a standard model for investigating chromosome behaviour *in silico* and for inferring chromosome properties from *in vivo* observations [26], [62].

In cell conditions, however, chromosomes are confined to move within the nucleus and by various structures contained therein. Additionally, at various stages throughout the cell-cycle and meiosis, chromosomes are observed tethered at, or close to, the inner nuclear surface rendering WLC treatments analytically intractable. Despite this intractability, useful properties may still be estimated for confined WLCs by discretising the chromosome into a series of loci, 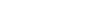, connected via inextensible rods, and adopting a sample-based approach. In these coarse-grained representations the chromosome is described in terms of the three-dimensional positions of its N loci, 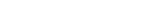 and by the inextensible rods that connect them, 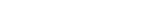, which may be related via:
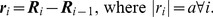
(1)The constraint, 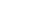, ensures the inextensibility of the ith rod, and may be set using 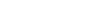, with 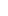 denoting the compaction-factor and 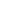 the fully-extended contour-length of the chromosome. The statistical behaviour under steady-state conditions is calculated by considering the energy associated with a particular configuration, 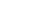, and assuming a Boltzmann distribution:
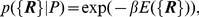
(2)where 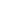 denotes the thermodynamic beta and 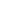 any free parameters used to define the energy. For spherically confined and/or tethered chromosomes this energy is calculated as:
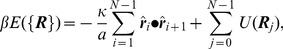
(3)where the first term represents the bending-energy associated with a particular configuration, 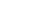 represents the bending-modulus of the chromosome (where 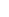 represents the persistence length), 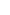 the unit vector of the ith rod and 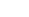 the dot-product between vectors 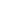 and 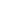. The second term in Equation (3) represents the confining potential imposed upon each locus by the nucleus (and other large nuclear structures) and is typically assumed to correspond to hard-core confinement. The vector, 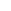, therefore contains the quintuple of parameters 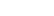 and 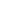. Additional terms may be included in Equation (3) to represent the fact that two loci cannot approach with a certain distance of one another (excluded-volume interaction) or approach within a certain distance of various nuclear structures. The inclusion of such terms, however, requires significantly more computation, and consequently were not included in our models.

Notable coarse-grained models include studies in which interphase chromosomes are modelled as spherically-confined worm-like chains with excluded-volume effects [63], and studies by [64], who used Markov chain Monte Carlo (MCMC) procedures to investigate the statistical behaviour of spherically-confined interphase chromosomes when either the centromere or telomeres were tethered at, or close to, the nuclear periphery. Other approaches infer chromosome structure from G1-phase measurement of cross-linking by assuming that chromosomes correspond to flexible polymers with no excluded-volume interactions [68], [83].

As far as we are aware no studies exist using coarse-grained, sample-based modeling of semiflexible chromosomes during meiosis, although some notable studies based upon scaling arguments exist [84], [85]

### Chromosome clustering forces

Besides chromosome tethering, a noticeable feature of nuclear architecture *in vivo*, particularly during meiosis, is the polarisation of chromosomes within the nucleus, wherein centromeres or telomeres are located to a limited region of the nuclear periphery. The polarisation of centromeres during early meiosis appears to require a degree of microfilament control [86]. Similarly, the (transient) polarisation of telomeres during the bouquet stage of meiosis appears to involve the directed motion of telomeres rather than random diffusion [46], with further studies identifying a nuclear-hugging microfilament network as the likely source of this biased motion [26]. Taken together these results suggest that clustering of centromere/telomeres over the nuclear periphery is actively enforced rather than an emergent property of confined and tethered polymers, and must therefore be explicitly incorporated into models of chromosome behaviour. This may be achieved by including additional terms in the systems energy, representing the force imposed upon centromere/telomeres by a microfilament network. For the case in which centromeres are tethered and experience polarising forces we write for the system energy:
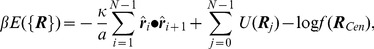
(4)where 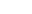 denotes the position of the centromere in space. The functional form of 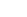 in Equation (4) is parameterised as
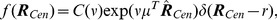
(5)which corresponds to the von Mises-Fisher (vMF) distribution with mean-vector, μ, and angular-variance (or spread of the distribution) ν. The von Mises-Fisher distribution represents a distribution over the surface of a sphere. When the angular variance ν = 0, samples from a vMF distribution will be uniformly distributed over the surface of the sphere, whilst increasingly positive values for ν will result in samples increasingly clustered on the surface about a mean vector, μ. Here 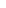 is chosen to correspond to the nuclear radius, and the constraint 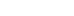, ensures the centromere always lies on the surface of the nuclear periphery. The effect of substituting Equation (5) into Equation (4) is, therefore, to cluster centromeres (over the nuclear periphery) about a mean vector, μ, with angular variance that depends upon both ν and emergent properties of the first two terms in Equation (4). Similarly, when telomeres are tethered to the nuclear periphery and clustered, the system's energy is calculated as:
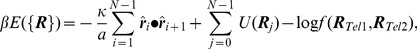
(6)where 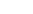 and 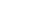 denote the positions of the first and second telomeres respectively. The functional form of 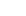 is chosen to correspond to a product of two independent von Mises-Fisher distributions:
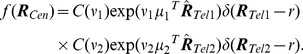
(7)It is important to note that within this model the vMF distributions (with positive, nonzero ν) only induce a clustering force upon either telomere and are not, in themselves, necessarily sufficient to induce clustering. The statistical behaviour of chromosome with polarising forces depends upon 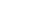 and emergent properties of the other terms in Equation (6). For short, rigid chromosomes, for example, the clustering forces will tend to want to induce a folding of the chromosome, whilst the internal rigidity of the chromosome will want to promote a straight trajectory, with the overall behaviour of the chromosome depending upon the relative magnitudes of these two effects. The probability density associated with a particular configuration, 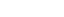, may be calculated by substitution of Equations (4) or (6) into (2). Whilst these distributions are analytically intractable, it is possible to sample representative trajectories by adopting an MCMC procedure similar to that used in [64].

### Parameter choice and Markov chain Monte Carlo sampling

The polymer-statistic models outlined above have been implemented in a Matlab toolbox Markov chain Monte Carlo (MCMC) for meiotic chromosomes (3MC) and used to investigate the influence of chromosome architecture upon locus proximity within *S. cerevisiae*. The 3MC package including front end graphical user interface (GUI) is available for download at http://wsbc.warwick.ac.uk/software/3MC/3MC.zip

In all subsequent models, the nuclear diameter was set to 2 µm in accordance with previous observations of nuclear diameter in *S.cerevisiae* [66], [67], with the spindle pole body (SPB) aligned along the positive z-axis ([0, 0, 1000] nm). Within the model the SPB has no physical influence on chromosome trajectories, but is used to set the direction of the centromere/telomere clustering i.e., the mean parameter, μ, in the von Mises-Fisher distribution(s) are aligned to the SPB. Currently, four different levels of clustering have been implemented, as defined by the angular variance parameter, ν. These values were set by eye, by observing the level of clustering induced on independent samples from the corresponding von Mises Fisher distribution. Values ranged from ν = 0, representing chromosomes in which centromere/telomeres are uniformly distributed over the entire nuclear surface [64], through weak (ν = 5), intermediate (ν = 20), and strong (ν = 50), representing the case in which centromeres/telomeres experience a strong force acting to cluster them about the SPB.

Three difference chromosome sizes were simulated, with the shortest modelled on yeast chromosome I (∼240 Kb), chromosome XVI (∼950 Kb) and chromosome IV (∼1530 Kb). The three chromosomes were assumed to correspond to the 30-nm fibre [62], [83], resulting in an approximately 40-fold reduction in contour length compared to dsDNA [87]. Sample chromosome trajectories were generated using 3MC, with chromosomes represented as 300-locus WLCs with centromeres located at an appropriate distance for the chromosome lengths tested. The choice to discretise into 300 beads represented a trade-off between the ideal number (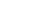) and computational time. Specifically, in the ideal case the chromosome would be divided into an infinite number of segments at which point the continuous WLC and discrete models become equivalent. In practice, however, the statistical behaviour of the model was found to converge very rapidly as the number of links increased, and the choice of 300 loci was found to be a good approximation to 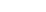 for all chromosome sizes tested (e.g. Fig. S8). Additionally, the choice of 300 segments meant that, for 40-fold compaction, even the longest chromosome would be divided into approximately 40-nm segments (close to the width of the 30-nm fibre) whilst being sufficiently small enough to allow calculations to be performed on desktop computers in a reasonable time. The persistence length was varied over 2 increments in the range 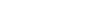, capturing the behaviour of flexible and semiflexible regimes. The above range of values was chosen to cover the range of previous values inferred from experimental measurements, with the lesser value corresponding to that for interphase chromosomes [62], and the more rigid value lying somewhere between this value and that observed for pachytene chromosomes [44].

In total, 10 million sample chromosome trajectories were generated for each model condition using MCMC procedures (the 3MC package), with the first 3 million samples discarded for burn-in. The remaining 7 million samples were thinned by a factor of 40, with the remaining samples used to empirically calculate the desired statistics, including the physical distance between allelic loci and between different loci on the same chromosome or on heterologous chromosomes.

### Model limitations

In order to allow investigation of a large range of chromosome architectures as well as a large range of model parameters, a number of simplifying assumptions were made. Specifically, the chromosomes were treated as line-like objects by ignoring the effects of excluded-volume interactions due to chromosome width/volume. In tests the influence of excluded volume (intrachromosomal only) was found to have negligible influence on the chromosome trajectories modeled in isolation (Fig. S9). This would not be the case if persistence lengths were very short (e.g 30 nm; data not shown). *In vivo* the volume of unmodeled chromosomes and subnuclear structures creating prohibited areas [17], [65], [74] would have an influence on the measured trajectories. Consequently, our results represent a first look into the effect of tethering and clustering during meiosis, and provide a good foundation for future studies that will include excluded volumes from other chromosomes. The influence of external volume has previously been included in coarse-grained models of interphase chromosomes [63]. With well defined parameters, excluded volumes can be incorporated in future models using additional terms in Equations (3) or (4).

## Supporting Information

Figure S1 **Sample trajectories for chromosome I homologues with centromeres tethered.** Flexible means persistence length 0.2 µm and rigid means persistence length 2.0 µm, nu refers to the clustering parameter ν (see Materials and Methods).
(BZ2)

Figure S2 **Sample trajectories for chromosome I homologues with telomeres tethered.** Conditions are as described for Fig. S1.
(BZ2)

Figure S3 **Sample trajectories for chromosome XVI homologues with centromeres tethered.** Conditions are as described for Fig. S1.
(BZ2)

Figure S4 **Sample trajectories for chromosome XVI homologues with telomeres tethered.** Conditions are as described for Fig. S1.
(BZ2)

Figure S5 **Sample trajectories for chromosome IV homologues with centromeres tethered.** Conditions are as described for Fig. S1.
(BZ2)

Figure S6 **Sample trajectories for chromosome IV homologues with telomeres tethered.** Conditions are as described for Fig. S1.
(BZ2)

Figure S7 **Sample trajectories for untethered chromosomes.** Conditions are as described for Fig. S1.
(BZ2)

Figure S8 **Convergence of measured statistics to the continuous wormlike chain (WLC).** In the above graphs the average pairwise distance between loci (averaged over all loci on the same chromosome and 1000 sample trajectories) is indicated on the Y-axis, plotted as a function of the discretisation number (X-axis; the number of segments the chromosome is divided into). Separate points for the same N correspond to different sets of 1000 sample trajectories. Chromosomes correspond to chromosome I (left) and IV (right), with persistence length 0.2 µm and both telomeres tethered to the nuclear periphery but otherwise unclustered (ν = 0; nuclear diameter = 2 µm). When N>75, further increasing the discretisation number yields little difference to estimated values, suggesting that the behaviour is a good approximation to the continuous WLC. In order to ensure good approximation to the continuous WLC we chose to represent the chromosome with N = 300 segments. Here we have used chromosomes with telomeres tethered to the nuclear periphery (ν = 0) as an example to illustrate convergence.
(TIF)

Figure S9 **The influence of excluded volume terms (within chromosome only) was found to have negligible influence on chromosome trajectories.** In particular, the excluded volume terms were modeled as an additional (repulsive) potential between all pairwise loci in the same chromosome with magnitude exp(100) if those loci approached within 40 nm of one another and zero otherwise. The influence of excluded volume interactions from the same chromosome should be greatest when chromosomes were more flexible (and therefore likely to fold back upon themselves) and under tight Bouquet conditions. For chromosome I with persistence length 200 nm under Tight Bouquet conditions (ν = 50) these terms were found to minimally influence the distribution of intrachromosomal distances (over all loci and a 1000 trajectories). These results arise because, for the choice of chromosome persistence lengths, chromosomes are unlikely to bring distal loci close enough where self-avoiding terms to arise. For very flexible chromosomes (e.g., freely jointed chains) these terms will become increasingly important. Our tests indicate that excluded volume terms within a chromosome become important with very short persistence lengths e.g. 30 nm (not shown). In light of these results and our choice of parameters, approximating chromosomes as volume-less lines was considered to be appropriate to the modeling framework.
(TIF)
