# Supplementary material for: Modeling Meiotic Chromosomes Indicates a Size Dependent Contribution of Telomere Clustering and Chromosome Rigidity to Homologue Juxtaposition
Source: PLoS Comput Biol. 2012 May 3;8(5):e1002496. doi: 10.1371/journal.pcbi.1002496 (PMC3342934; doi:10.1371/journal.pcbi.1002496)

Chr. 4, telomeres tethered, nu=0, flexible

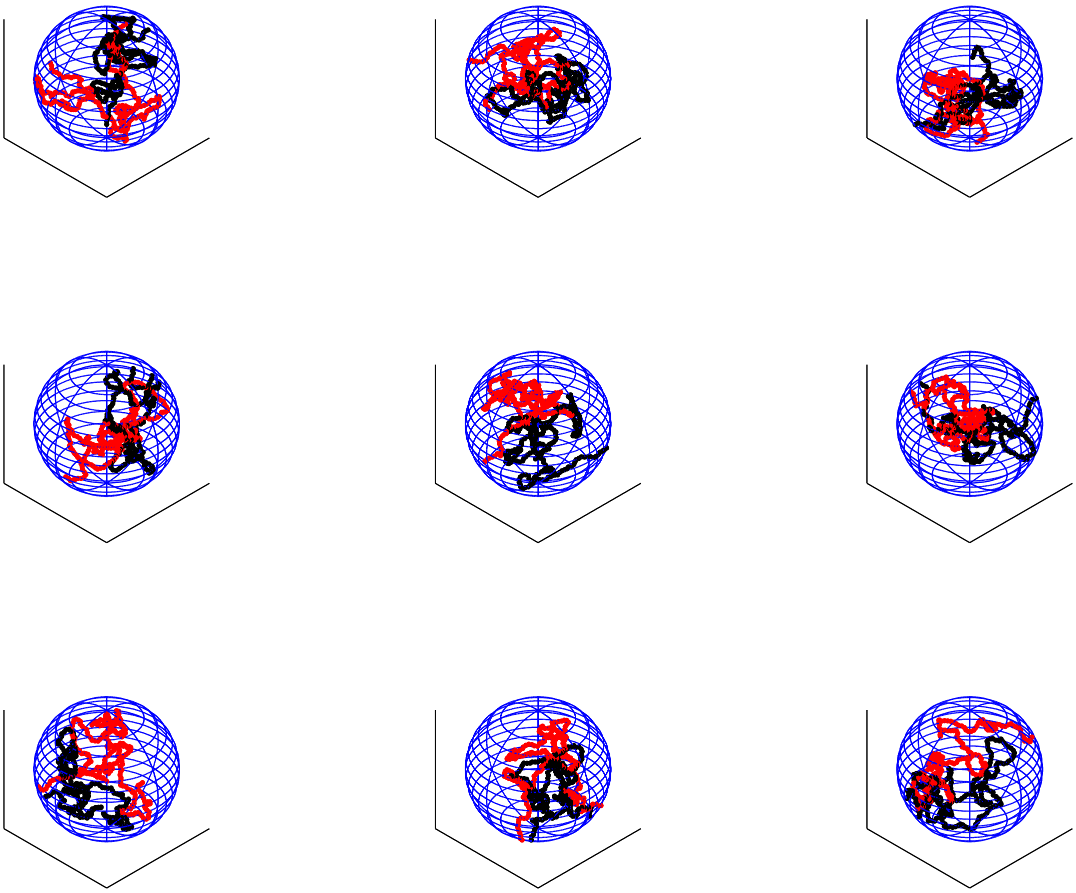

Chr. 4, telomeres tethered, nu=0, rigid

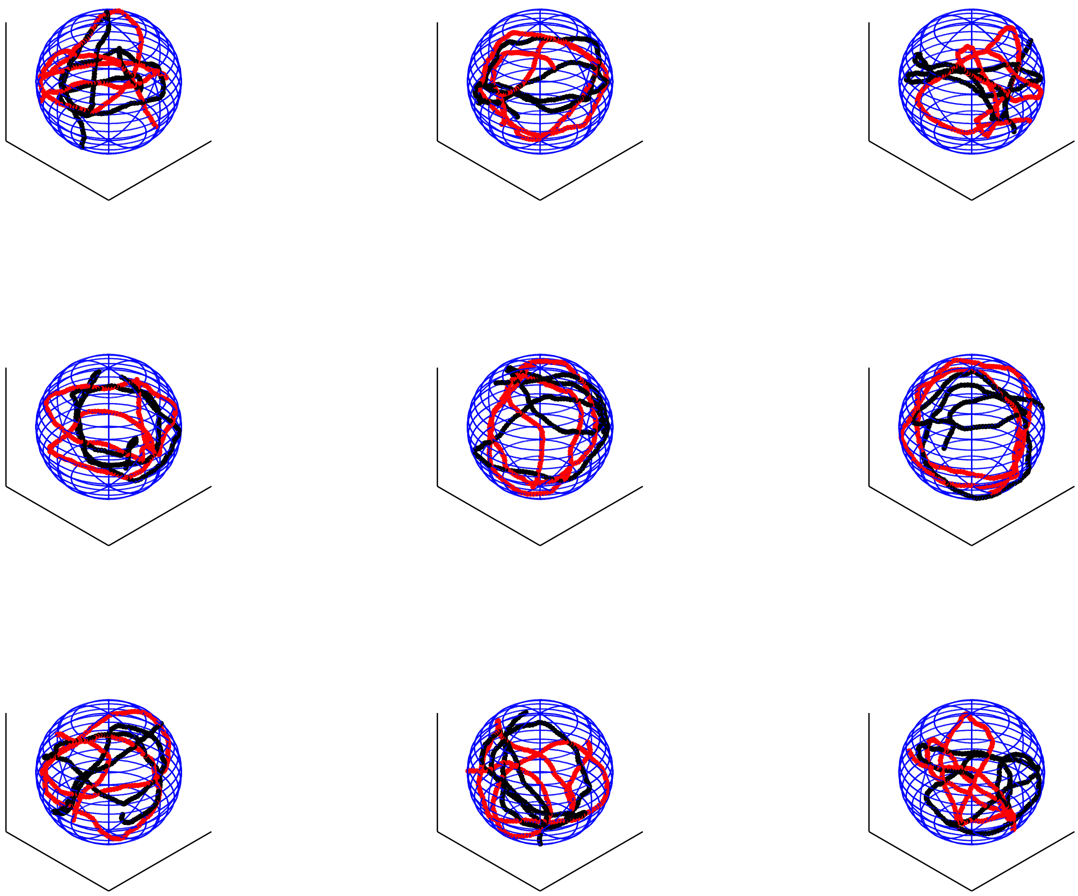

Chr. 4, telomeres tethered, nu=5, flexible

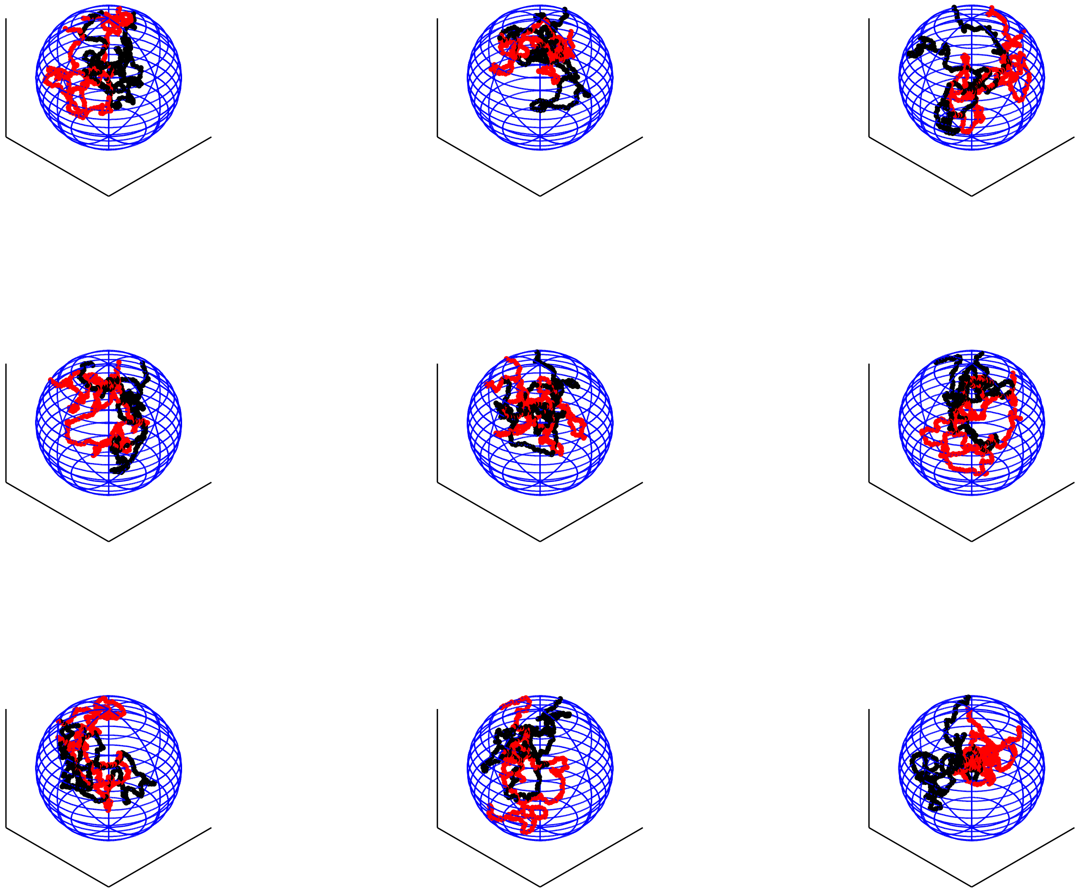

Chr. 4, telomeres tethered, nu=5, rigid

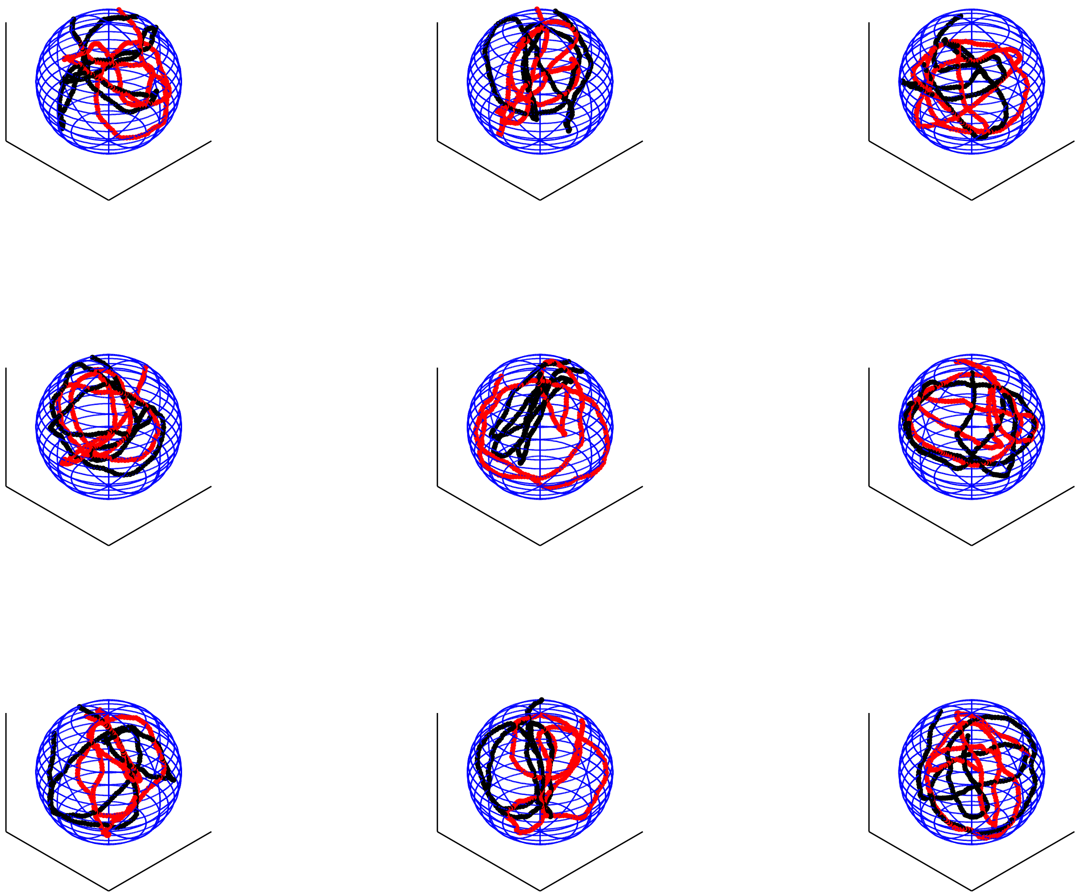

Chr. 4, telomeres tethered, nu=20, flexible

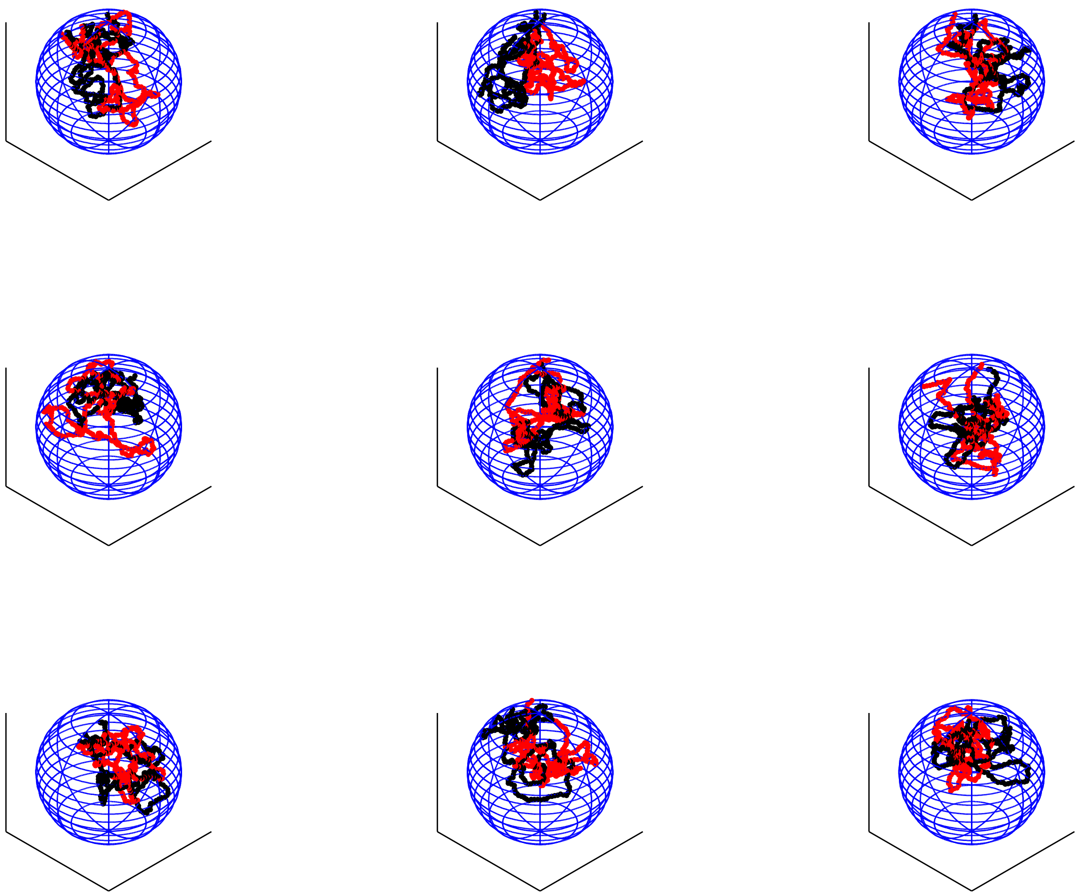

Chr. 4, telomeres tethered, nu=20, rigid

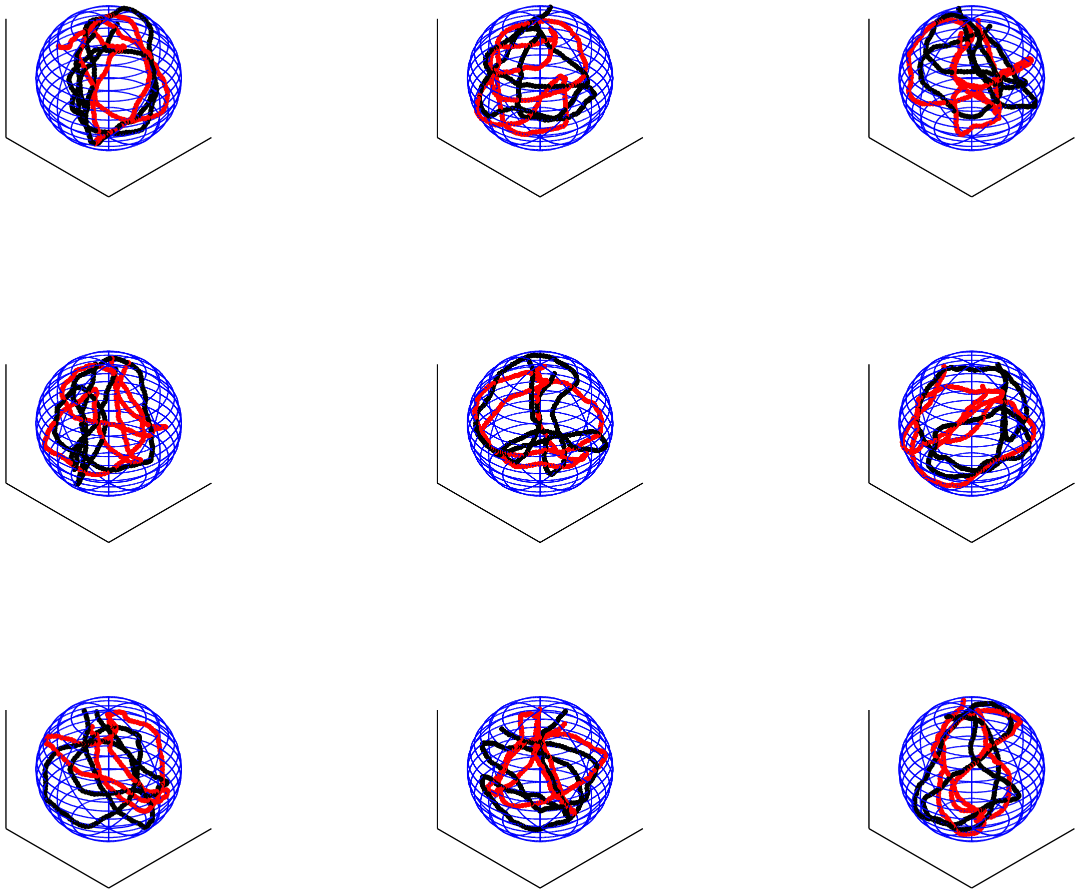

Chr. 4, telomeres tethered, nu=50, flexible

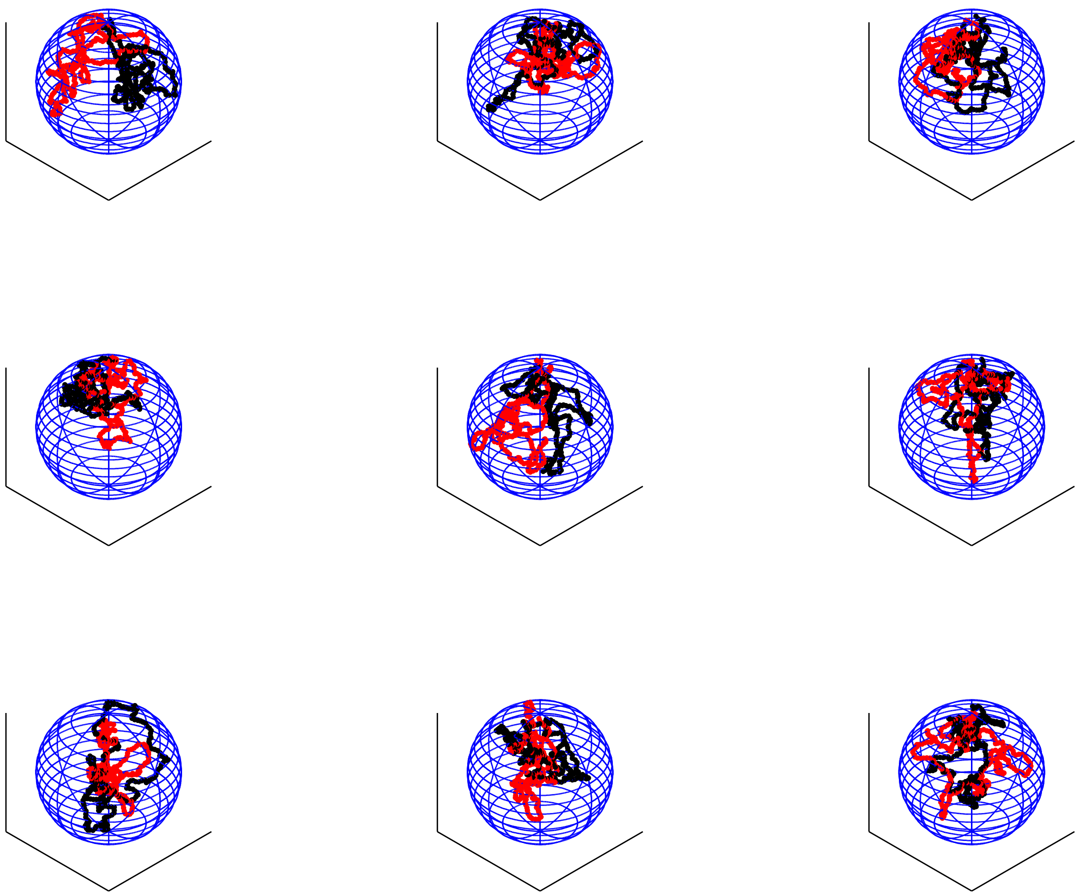

Chr. 4, telomeres tethered, nu=50, rigid

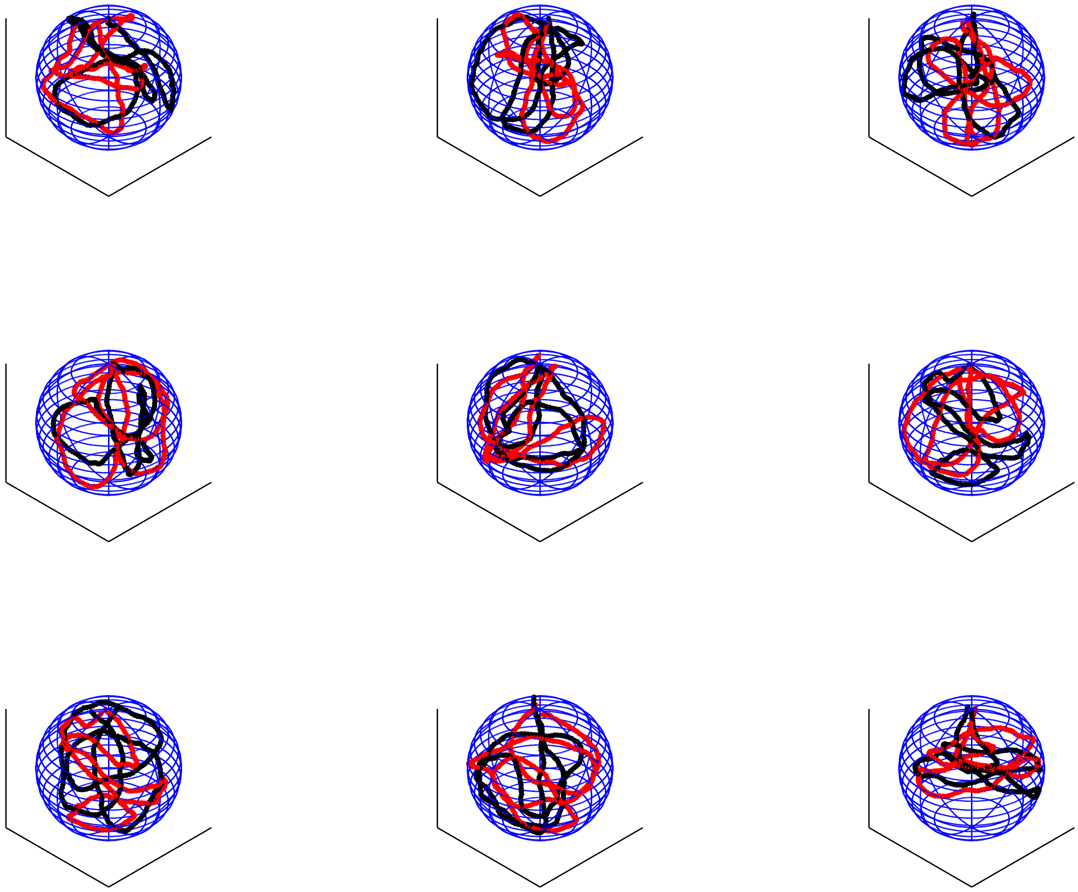

Supplement: Figure S6 — Sample trajectories for chromosome IV homologues with telomeres tethered. Conditions are as described for Fig. S1. (BZ2) [file pcbi.1002496.s006.bz2 › pcbi.1002496.s006]
