# Supplementary material for: Modeling Meiotic Chromosomes Indicates a Size Dependent Contribution of Telomere Clustering and Chromosome Rigidity to Homologue Juxtaposition
Source: PLoS Comput Biol. 2012 May 3;8(5):e1002496. doi: 10.1371/journal.pcbi.1002496 (PMC3342934; doi:10.1371/journal.pcbi.1002496)

Chr. 1, untethered, flexible

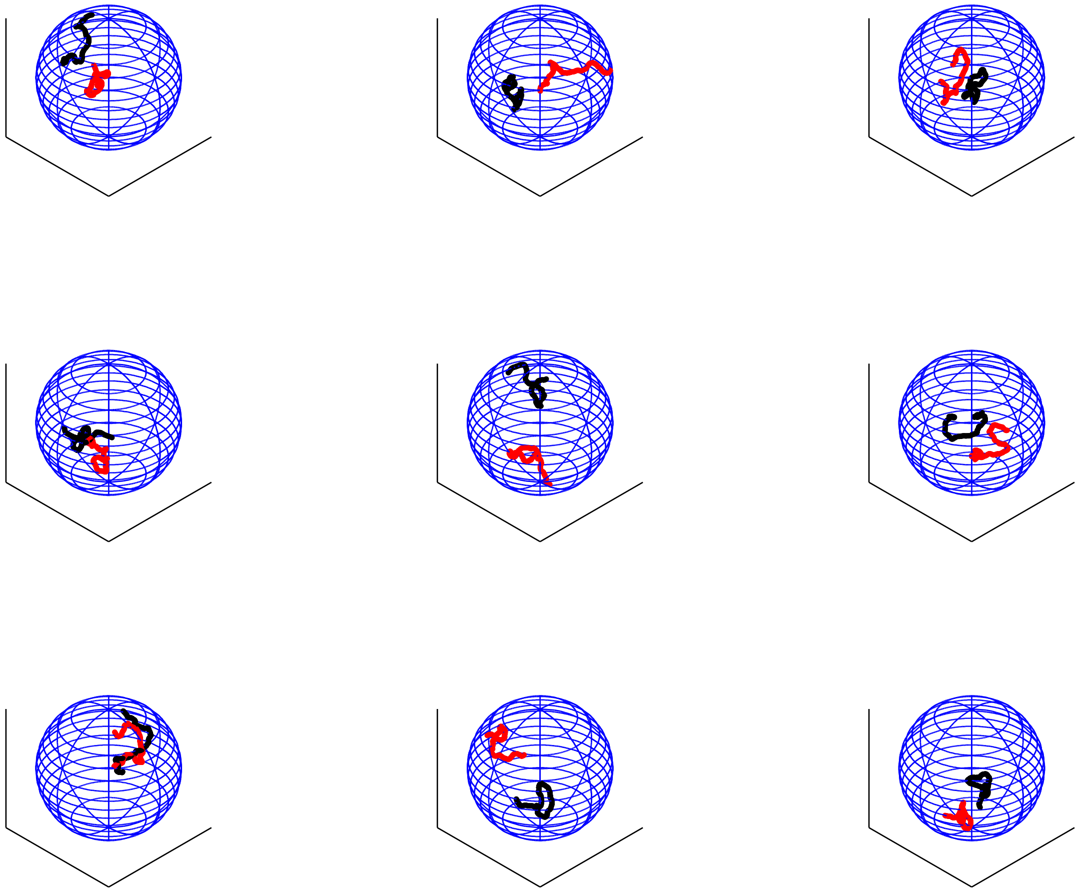

Chr. 1, untethered, rigid

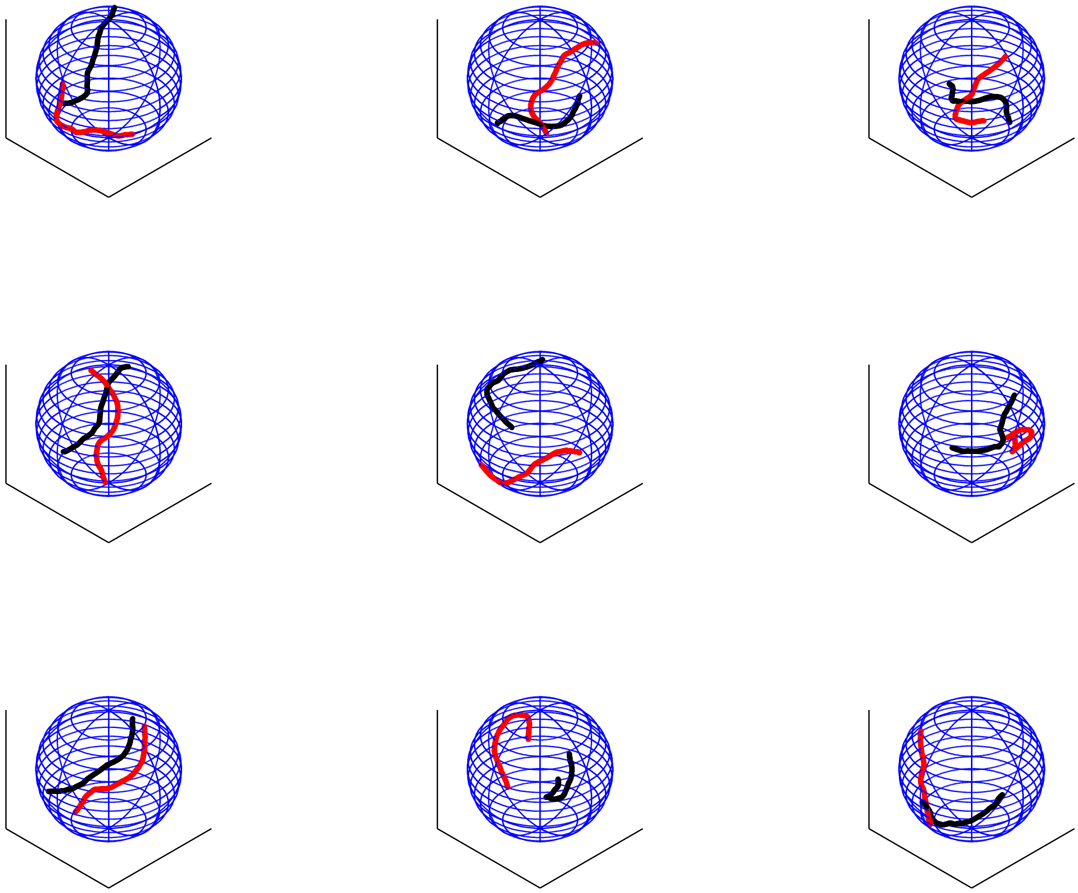

Chr. 16, untethered, flexible

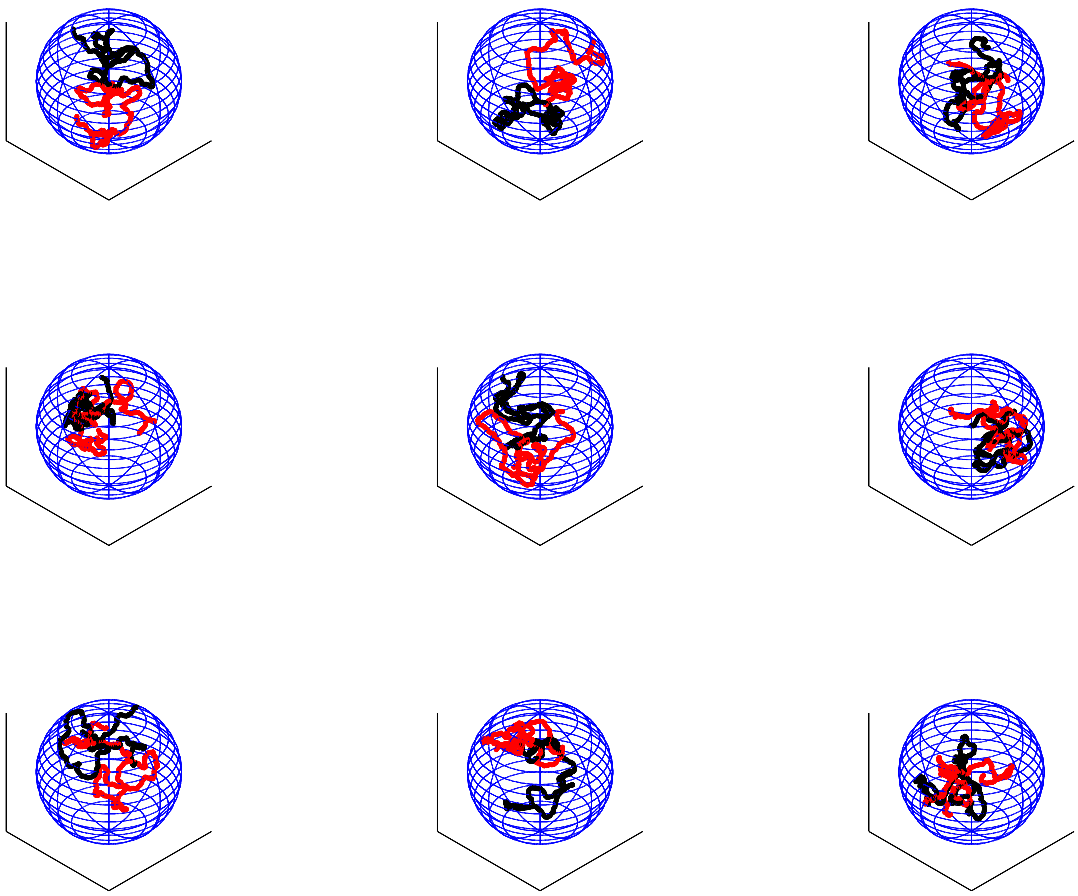

Chr. 16, untethered, rigid

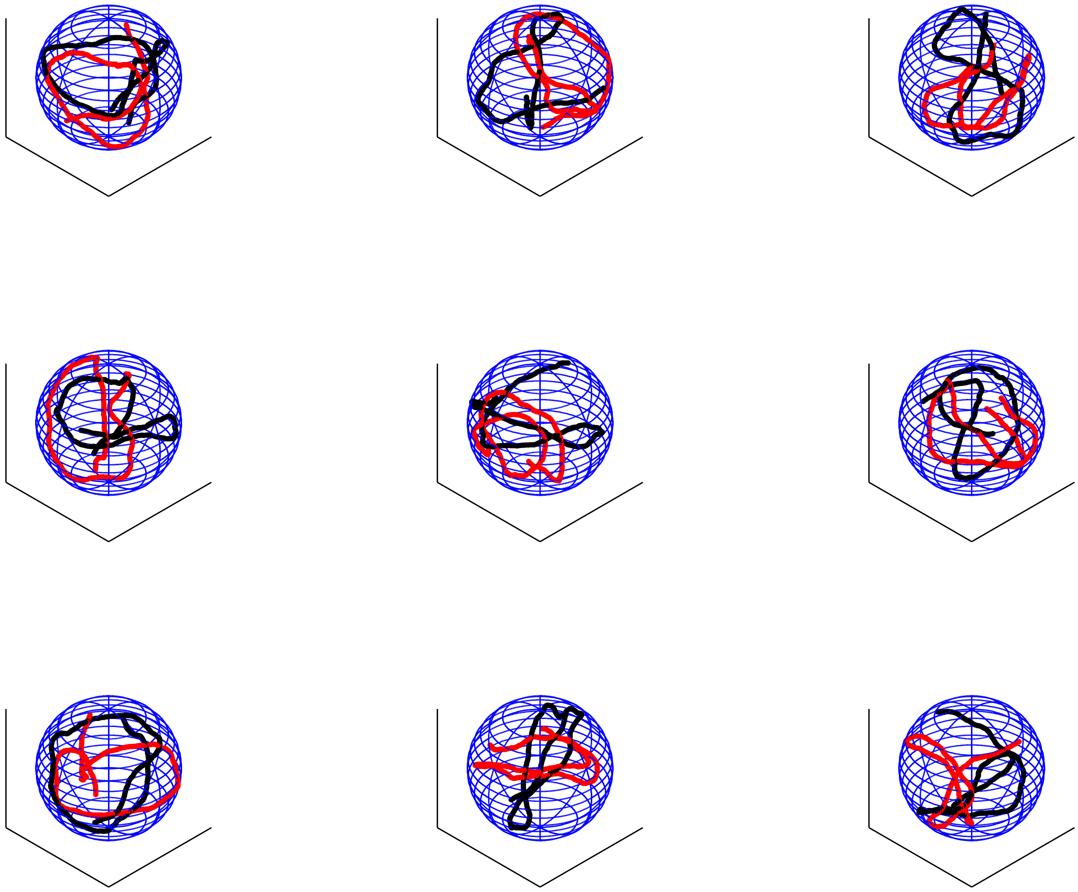

Chr. 4, untethered, flexible

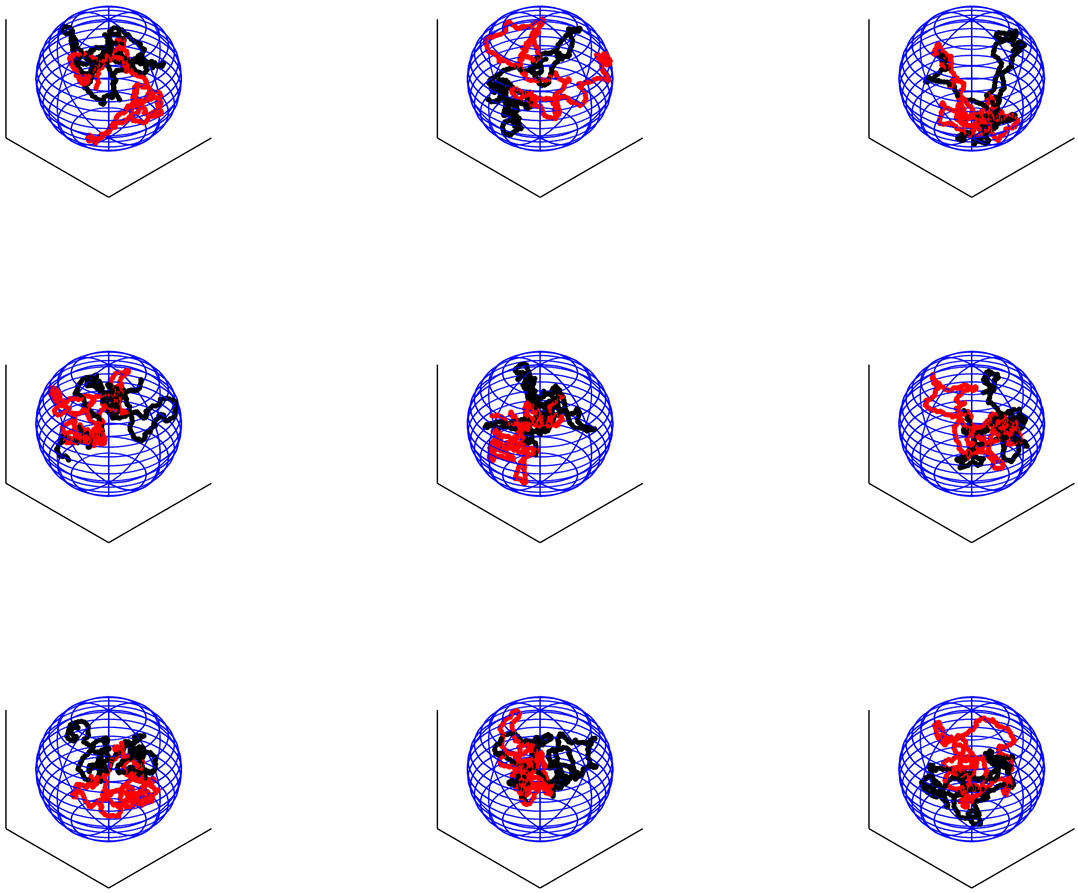

Chr. 4, untethered, rigid

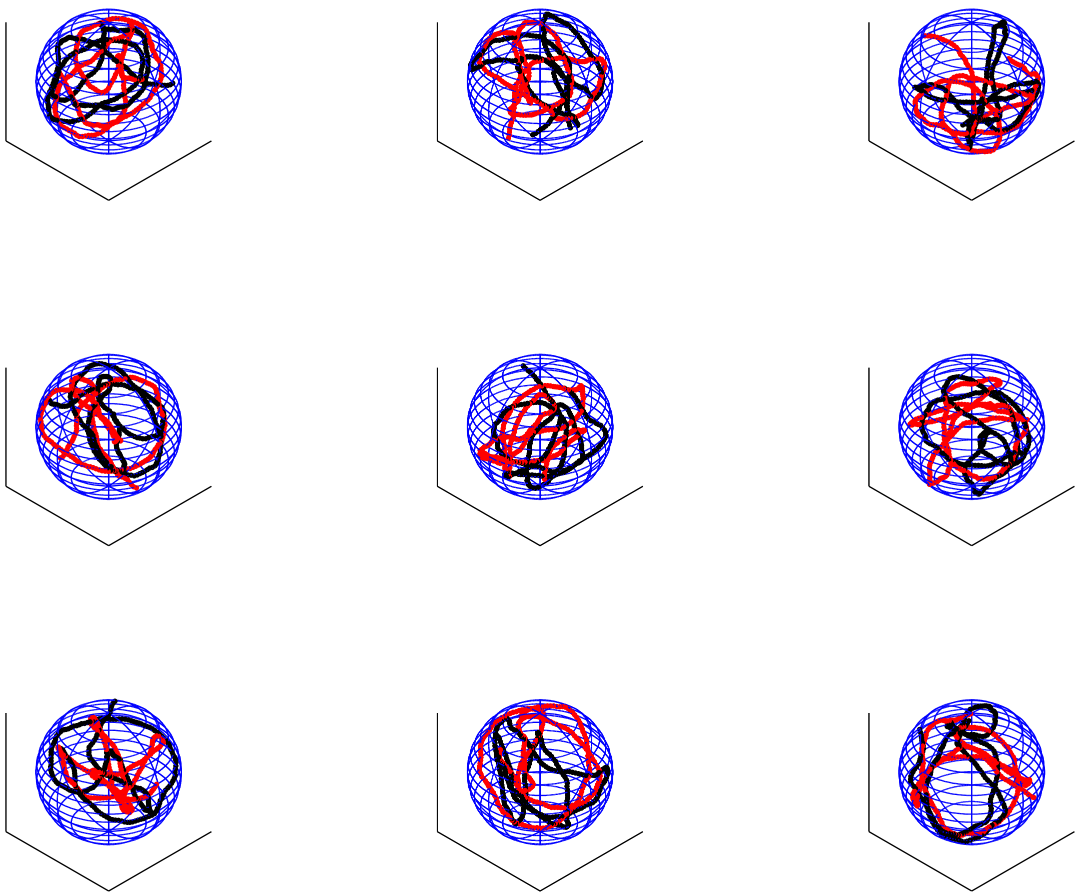

Supplement: Figure S7 — Sample trajectories for untethered chromosomes. Conditions are as described for Fig. S1. (BZ2) [file pcbi.1002496.s007.bz2 › pcbi.1002496.s007]
