# Supplementary figures and images for: Modeling Meiotic Chromosomes Indicates a Size Dependent Contribution of Telomere Clustering and Chromosome Rigidity to Homologue Juxtaposition
Source: PLoS Comput Biol. 2012 May 3;8(5):e1002496. doi: 10.1371/journal.pcbi.1002496 (PMC3342934; doi:10.1371/journal.pcbi.1002496)

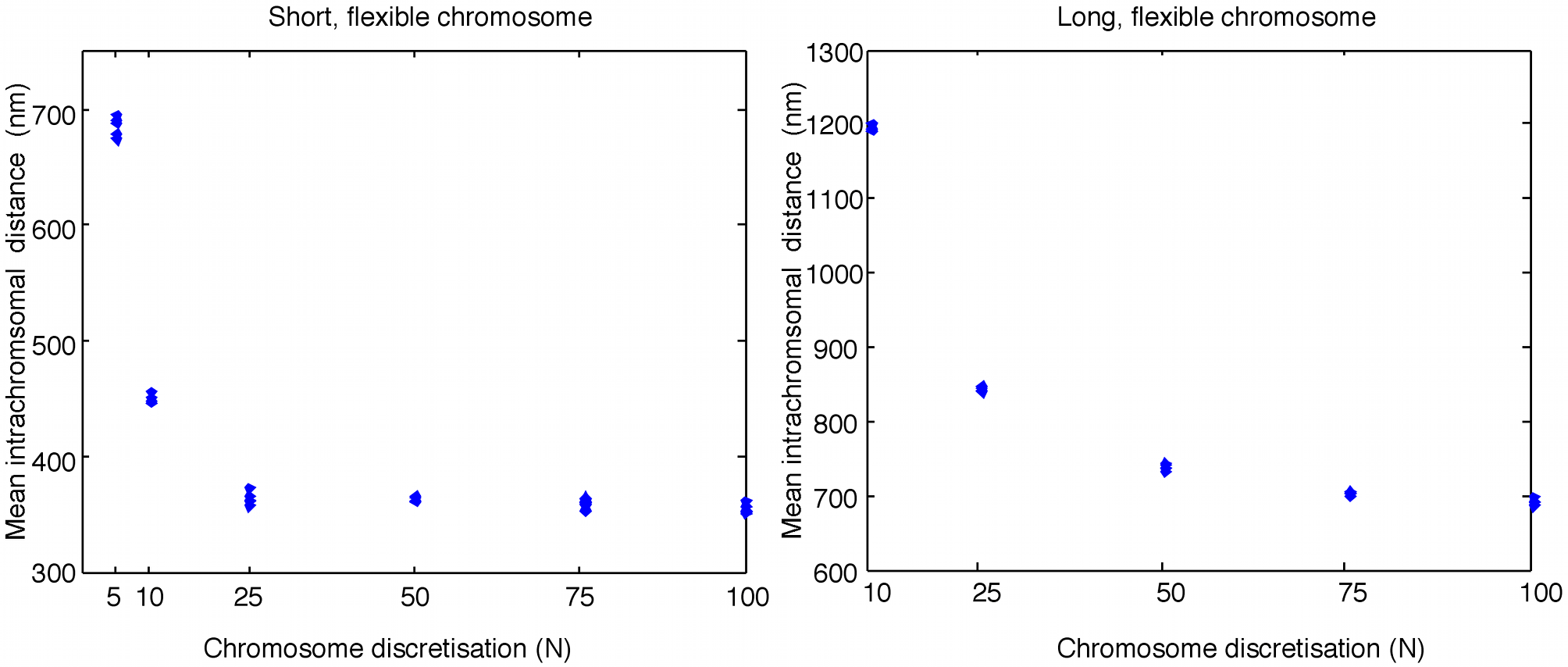

Supplement: Figure S8 — Convergence of measured statistics to the continuous wormlike chain (WLC). In the above graphs the average pairwise distance between loci (averaged over all loci on the same chromosome and 1000 sample trajectories) is indicated on the Y-axis, plotted as a function of the discretisation number (X-axis; the number of segments the chromosome is divided into). Separate points for the same N correspond to different sets of 1000 sample trajectories. Chromosomes correspond to chromosome I (left) and IV (right), with persistence length 0.2 µm and both telomeres tethered to the nuclear periphery but otherwise unclustered (ν = 0; nuclear diameter = 2 µm). When N>75, further increasing the discretisation number yields little difference to estimated values, suggesting that the behaviour is a good approximation to the continuous WLC. In order to ensure good approximation to the continuous WLC we chose to represent the chromosome with N = 300 segments. Here we have used chromosomes with telomeres tethered to the nuclear periphery (ν = 0) as an example to illustrate convergence. (TIF) [file pcbi.1002496.s008.tif]

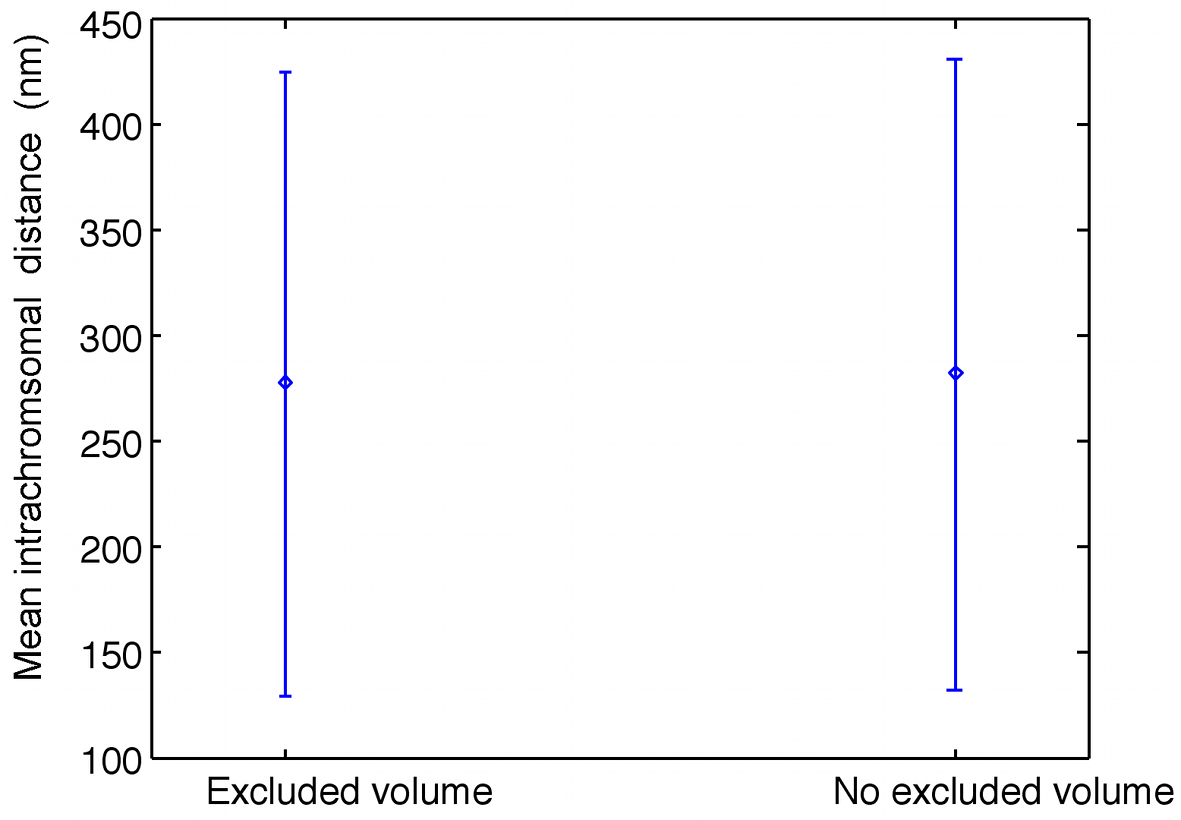

Supplement: Figure S9 — The influence of excluded volume terms (within chromosome only) was found to have negligible influence on chromosome trajectories. In particular, the excluded volume terms were modeled as an additional (repulsive) potential between all pairwise loci in the same chromosome with magnitude exp(100) if those loci approached within 40 nm of one another and zero otherwise. The influence of excluded volume interactions from the same chromosome should be greatest when chromosomes were more flexible (and therefore likely to fold back upon themselves) and under tight Bouquet conditions. For chromosome I with persistence length 200 nm under Tight Bouquet conditions (ν = 50) these terms were found to minimally influence the distribution of intrachromosomal distances (over all loci and a 1000 trajectories). These results arise because, for the choice of chromosome persistence lengths, chromosomes are unlikely to bring distal loci close enough where self-avoiding terms to arise. For very flexible chromosomes (e.g., freely jointed chains) these terms will become increasingly important. Our tests indicate that excluded volume terms within a chromosome become important with very short persistence lengths e.g. 30 nm (not shown). In light of these results and our choice of parameters, approximating chromosomes as volume-less lines was considered to be appropriate to the modeling framework. (TIF) [file pcbi.1002496.s009.tif]
